# Neighborhood-Level Nitrogen Dioxide Inequalities Contribute to Surface Ozone Variability in Houston, Texas

**DOI:** 10.1021/acsestair.4c00009

**Published:** 2024-07-30

**Authors:** Isabella M. Dressel, Sixuan Zhang, Mary Angelique G. Demetillo, Shan Yu, Kimberly Fields, Laura M. Judd, Caroline R. Nowlan, Kang Sun, Alexander Kotsakis, Alexander J. Turner, Sally E. Pusede

**Affiliations:** † Department of Environmental Sciences, University of Virginia, Charlottesville, Virginia 22904, United States; ‡ NASA Langley Research Center, Hampton, Virginia 23681, United States; § Department of Statistics, University of Virginia, Charlottesville, Virginia 22904, United States; ∥ Carter G. Woodson Institute for African American and African Studies, University of Virginia, Charlottesville, Virginia 22904, United States; ⊥ Atomic and Molecular Physics Division, Center for Astrophysics | Harvard & Smithsonian, Cambridge, Massachusetts 02138, United States; # Department of Civil, Structural and Environmental Engineering, University at Buffalo, Buffalo, New York 14260, United States; ○ Research and Education in eNergy, Environment and Water (RENEW) Institute, University at Buffalo, Buffalo, New York 14260, United States; ◆ NASA Goddard Space Flight Center, Greenbelt, Maryland 20771, United States; ∇ Department of Atmospheric Sciences, University of Washington, Seattle, Washington 98195, United States

**Keywords:** Nitrogen dioxide, ozone, TROPOMI, urban air pollution, environmental racism

## Abstract

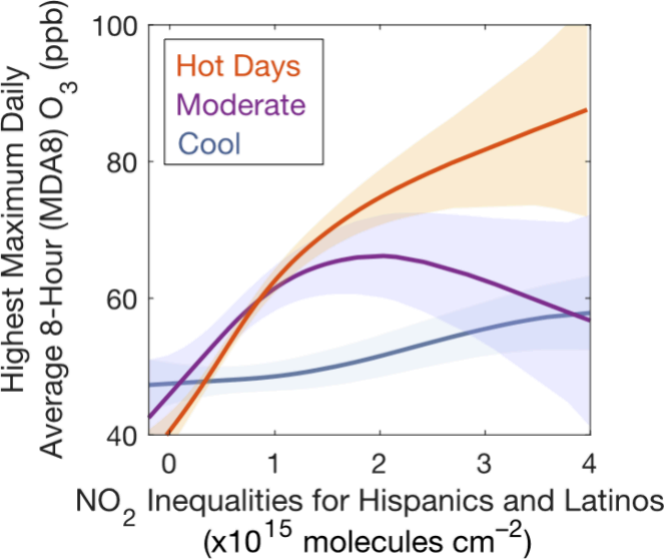

In Houston, Texas, nitrogen dioxide (NO_2_) air pollution disproportionately affects Black, Latinx, and Asian communities, and high ozone (O_3_) days are frequent. There is limited knowledge of how NO_2_ inequalities vary in urban air quality contexts, in part from the lack of time-varying neighborhood-level NO_2_ measurements. First, we demonstrate that daily TROPOspheric Monitoring Instrument (TROPOMI) NO_2_ tropospheric vertical column densities (TVCDs) resolve a major portion of census tract-scale NO_2_ inequalities in Houston, comparing NO_2_ inequalities based on TROPOMI TVCDs and spatiotemporally coincident airborne remote sensing (250 m × 560 m) from the NASA TRacking Aerosol Convection ExpeRiment–Air Quality (TRACER-AQ). We further evaluate the application of daily TROPOMI TVCDs to census tract-scale NO_2_ inequalities (May 2018–November 2022). This includes explaining differences between mean daily NO_2_ inequalities and those based on TVCDs oversampled to 0.01° × 0.01° and showing daily NO_2_ column-surface relationships weaken as a function of observation separation distance. Second, census tract-scale NO_2_ inequalities, city-wide high O_3_, and mesoscale airflows are found to covary using principal component and cluster analysis. A generalized additive model of O_3_ mixing ratios versus NO_2_ inequalities reproduces established nonlinear relationships between O_3_ production and NO_2_ concentrations, providing observational evidence that neighborhood-level NO_2_ inequalities and O_3_ are coupled. Consequently, emissions controls specifically in Black, Latinx, and Asian communities will have co-benefits, reducing both NO_2_ disparities and high O_3_ days city wide.

## Abstract 2

Most neighborhood-level NO_2_ inequalities can be observed with daily TROPOspheric Monitoring Instrument (TROPOMI) observations; the unequal NO_2_ distribution affects O_3_ chemistry in Houston, Texas.

## Introduction

Houston, Texas is a large U.S. city and center for petrochemical refining that faces multiple air quality challenges. Historical and contemporary policies and practices continue to disproportionately offload the environmental costs of industry and transportation on Black, Latinx, and Asian communities,^1,2^ causing measurable inequalities in the distribution of nitrogen dioxide (NO_2_) and other primary pollutants.^3−9^ Houston is also currently ranked among the top-ten most ozone (O_3_) polluted cities in the U.S., with residents experiencing frequent exceedances of health-based O_3_ standards city wide.^10^ Recent analytical advances have produced more spatially detailed descriptions of neighborhood-level urban air pollution inequalities,^11−15^ including for NO_2_.^16−18^ However, enhanced spatial information has generally relied on time-averaged and/or short-duration observations, representing conditions that potentially infrequently occur and limiting our understanding of relationships between NO_2_ inequalities and broader urban air quality issues such as O_3_. This has policy relevance as states have regulatory authority around O_3_ compliance that they often lack or decline to use regarding air pollution environmental injustice.

**Figure 1:**
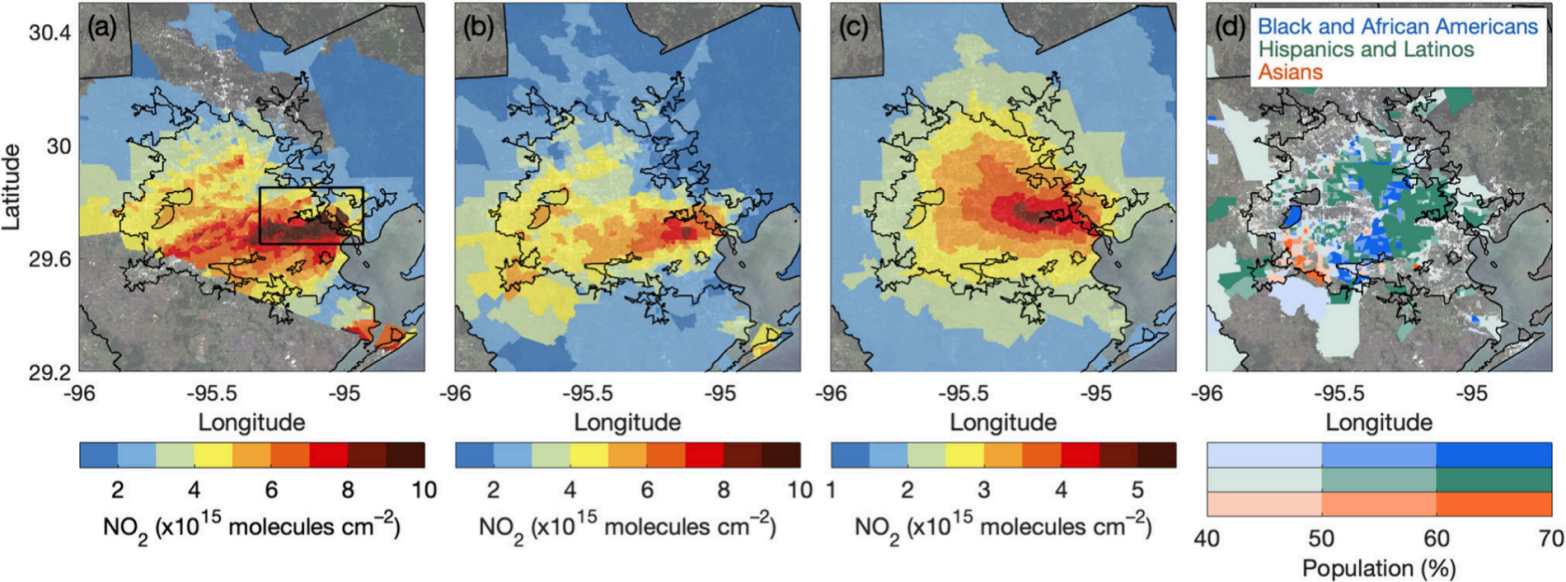
Example of census tract-scale GCAS NO_2_ columns (molecules cm^–2^) collected on 25 September 2021 at 2–5 pm (a), TROPOMI TVCDs on the same day, with a mean pixel size of 21 ± 0.6 km^2^ (b), and oversampled TROPOMI TVCDs (0.01° × 0.01°) over May 2018–November 2022 (c). Also shown, the percent population for the largest race-ethnicity group in each census tract for Black and African Americans (blue), Hispanics and Latinos (green), and Asians (orange) (d). The inner and outer black lines are the Urbanized Area (UA) and Metropolitan Statistical Area (MSA) boundaries, respectively. The thick black box is the Houston Ship Channel (a). Background map data: Landsat 8 composite (January 2017–June 2018). Corresponding wind conditions are presented in Figure S1.

NO_2_ is a criteria pollutant regulated by the U.S. Environmental Protection Agency (EPA). NO_2_ is a primary pollutant (or pseudo-primary pollutant) with a summertime atmospheric lifetime as short as a few hours. Primary pollutants are highly spatiotemporally variable, exhibiting atmospheric dispersion gradients of hundreds of meters to 1–2 km.^11,19,20^ NO_2_ is emitted as NO_*x*_ (≡ NO + NO_2_), with vehicles and electricity generation being major NO_*x*_ sources in U.S. cities.^21−23^ Houston is also a global hub for petrochemical manufacturing, where refineries and industrial activities contribute a large portion of NO_*x*_ emissions,^24−26^ especially in the Houston Ship Channel,^24−26^ a residential and industrial area along the Buffalo Bayou River, connecting downtown to Galveston Bay and the Gulf of Mexico (Figure 1). Associated with numerous adverse^27−31^ and unequal health impacts,^28^ NO_2_ is a common proxy for toxic combustion and traffic air pollution mixtures in health studies.^32^ High-volume roadways and heavy-duty diesel truck traffic overburden communities of color,^33,34^ and living near roadways is linked to asthma-related urgent medical visits, pediatric asthma, preeclampsia and preterm birth, and cardiac and pulmonary mortality.^35−40^

Neighborhood-level NO_2_ inequalities with race and ethnicity can be observed from space using the TROPOspheric Monitoring Instrument (TROPOMI).^3,16,41−45^ This was first demonstrated by Demetillo et al.,^3^ who showed relative census tract-scale NO_2_ inequalities based on TROPOMI tropospheric vertical column densities (TVCDs) oversampled to 0.01° × 0.01° agreed with results from fine-scale (250 m × 500 m) airborne remote sensing during the NASA Deriving Information on Surface Conditions from COlumn and VERtically Resolved Observations Relevant to Air Quality (DISCOVER-AQ) in Houston. In addition, spatial patterns in oversampled TROPOMI TVCDs reflected NO_2_ distributions at the surface, a conclusion based on comparisons with in-situ aircraft NO_2_ vertical profiles from DISCOVER-AQ and surface measurements.^3^ In a subsequent analysis of 52 U.S. cities, Demetillo et al.^16^ reported oversampled TROPOMI NO_2_ inequalities were invariant with urban racial segregation structure,^34^ meaning that TROPOMI resolves inter-tract NO_2_ differences even when segregated tracts do not spatially aggregate into larger regions. Dressel et al.^41^ found mean daily TROPOMI observations (3.5 km × 5.5 km at nadir) without oversampling also captured a majority of tract-scale NO_2_ inequalities compared to fine-scale (250 m × 250 m) airborne remote sensing and agreed with relative NO_2_ inequalities based on TVCDs oversampled to 0.01° × 0.01° to within associated uncertainties, at least in New York City, New York and Newark, New Jersey. Daily NO_2_ inequalities, when uncertainties are well-characterized, can be analyzed statistically and situated within our broader understanding of urban air quality.^41^

NO_2_ is an O_3_ precursor and temporary O_3_ reservoir (O_*x*_ ≡ NO_2_ + O_3_), with O_3_ production chemistry varying nonlinearly with NO_2_ and the reactivity of volatile organic compounds (VOCs) with hydroxyl radical (OH). O_3_ pollution in Houston is attributed in large part to the combination of high NO_*x*_ and reactive VOC emissions by industries in the Ship Channel and gulf breeze airflows.^26,46−51^ While O_3_ air quality has improved,^52−54^ exceedances of the health-based maximum daily average 8-h (MDA8) O_3_ National Ambient Air Quality Standard (NAAQS) of 70 ppb are frequent, with 141 exceedance days in the Houston Metropolitan Statistical Area (MSA) over May 2018–November 2022 (our study period). O_3_ is a secondary and intermediately long-lived pollutant. As a result, O_3_ exhibits less intraurban heterogeneity than NO_2_ and is not generally associated with neighborhood-level disparities.^55^ However, because NO_2_ and VOC concentrations are spatiotemporally variable, O_3_ production (*P*O_3_) chemistry is as well,^56−58^ with NO_2_ inequalities and city-wide O_3_ potentially coupled. In Houston, the largest NO_2_ inequalities during DISCOVER-AQ corresponded to a severe O_3_ event with MDA8 O_3_ of 124 ppb (LaPorte Sylvan Beach, 25 September 2013).^3^ In New York City–Newark, tract-scale NO_2_ inequalities were positively associated with summertime MDA8 O_3_ (2018–2021), with Spearman correlation coefficients of 0.41–0.55 for different population groups.^41^

Here, we describe census tract-scale TROPOMI NO_2_ inequalities and investigate relationships with MDA8 O_3_ in Houston. As a first step, we evaluate daily TROPOMI NO_2_ inequalities with race-ethnicity, advancing our understanding of the application of mean daily TROPOMI NO_2_ TVCDs to NO_2_ inequalities developed in New York City–Newark.^41^ We compare daily TROPOMI NO_2_ inequalities against measurements of spatiotemporally coincident airborne remote sensing (250 m × 560 m) during the NASA TRacking Aerosol Convection ExpeRiment–Air Quality (TRACER-AQ) in September 2021, discuss differences between relative and absolute mean daily and oversampled TROPOMI NO_2_ inequalities, and present column-surface relationships as a function of measurement separation distance and surface wind conditions. Second, we statistically analyze TROPOMI NO_2_ inequalities (May 2018–November 2022), interpreting covariations between neighborhood-level NO_2_ inequalities, overall NO_2_ pollution, and urban O_3_ air quality in ways that have policy implications.

## Measurements and Methods

### TROPOMI

TROPOMI is a hyperspectral spectrometer onboard the sun-synchronous European Space Agency Copernicus Sentinel-5 Precursor (S-5P) satellite.^59,60^ NO_2_ is retrieved by fitting the 405–465 nm spectral band based on an updated Dutch OMI (Ozone Monitoring Instrument) NO_2_ (DOMINO) algorithm and work from the Quality Assurance for Essential Climate Variables project.^61−65^ NO_2_ observations are converted to TVCDs via an air mass factor (AMF), which relies on spatially and temporally coarse inputs, e.g., clouds, surface albedo, and NO_2_ profile shape, that can bias NO_2_ TVCDs low under high NO_2_ conditions.^66^ The application of TROPOMI NO_2_ TVCDs to census tract-scale NO_2_ inequalities has been evaluated through comparison with airborne remote sensing that resolves NO_2_ distance decay gradients, both in terms of TVCDs first oversampled to 0.01° × 0.01°^3^ and daily TVCDs,^41^ with TROPOMI capturing similar relative but lower absolute population-weighted census tract-scale NO_2_ inequalities. While the sensitivity of TROPOMI is lower near the surface,^67,68^ there are no physical processes in the free troposphere that maintain intraurban gradients corresponding to neighborhood-level race-ethnicity. TROPOMI TVCDs have been shown to reflect intraurban spatiotemporal NO_2_ variability at the surface, a critical analytical requirement for informing decision making around environmental racism.^3,16,41^ Based on 144 in-situ NO_2_ vertical profiles throughout Houston from DISCOVER-AQ, Demetillo et al.^3^ reported that the slope of the linear fit between the measured full column (extending up to 3 km) and NO_2_ column within the convective boundary layer was 0.98 ± 0.15 (*r* = 0.99), with no significant location-specific differences. Multiple authors have shown TROPOMI and OMI NO_2_ TVCDs correlate with surface-level nitrogen dioxide (NO_2_*) measurements and, more importantly, that correlation coefficients decrease with increasing spatial separation between columns and monitors on the scales of NO_2_ spatial variability.^3,16,41,69^

From 1 May 2018 to 5 August 2019, the TROPOMI nadir spatial resolution was 3.5 km × 7 km; from 6 August 2019 to present, the nadir spatial resolution improved to 3.5 km × 5.5 km.^70^ The S-5P satellite crosses the equator at ∼1:30 pm local time (LT) and overflies Houston at 12–3 pm LT, typically once but occasionally twice daily. When there are two TROPOMI overpasses over Houston on the same day, we use the first overflight only. We use current Level 2 NO_2_ TVCDs (version 02.04.00) with quality assurance values >0.75, as recommended,^71^ from operationally reprocessed (RPRO, collection identified: ‘03′, 1 May 2018–25 July 2022) and offline (OFFL, 26 July 2022–30 November 2022) products. A key update in version 02.04.00 is the use of a surface albedo climatology derived from TROPOMI observations rather than the coarse spatial resolution OMI surface albedo climatology (0.5° × 0.5°).^71^ TROPOMI NO_2_ inequalities can be sensitive to product version; for example, Dressel et al.^41^ found census tract-scale NO_2_ inequalities based on NO_2_ TVCDs reprocessed on the S-5P Products Algorithm Laboratory (S5P-PAL) system were 3–6 points (10–20%) higher over the New York City–Newark urbanized area (UA) than those computed using a then current version of operational product (version 01.02.02). We compared NO_2_ inequalities using version 02.04.00 (RPRO) and S5P-PAL reprocessed TVCDs over January–December 2019 but find results were statistically indistinguishable.

### GCAS

The Geostationary Coastal and Air Pollution Events (GEO-CAPE) Airborne Simulator (GCAS) makes hyperspectral nadir-looking measurements of backscattered solar radiation in the ultraviolet and visible in two channels at wavelengths 300–490 nm (optimized for air quality) and 480–900 nm (optimized for ocean color).^72^ Each channel uses a two-dimensional (2D) charge-coupled device (CCD) array detector, where one CCD dimension provides spectral coverage and the other the cross-track spatial coverage across a ∼45° field of view in the air quality channel. GCAS was developed as a technology-demonstration instrument for the GEOstationary Coastal and Air Pollution Events (GEO-CAPE) decadal survey and functions as a satellite analog in NASA airborne research. GCAS NO_2_ column retrievals are validated over urban areas and consist of a two-step approach similar to algorithms used for other major satellite instruments, including TROPOMI.^73−75^ Briefly, NO_2_ differential slant columns are retrieved fitting across 425–460 nm using the QDOAS spectral fitting package^76^ and a reference spectrum measured at a nearby location away from NO_*x*_ emissions sources. The AMF is largely a function of viewing and solar geometries, surface reflectance, and atmospheric and trace gas vertical profiles.^73,77^ GCAS retrievals for TRACER-AQ use the NASA GEOS-CF model analyses (0.25° × 0.25°).^78^ Other components of the retrieval follow Judd et al.,^77^ where column uncertainties over New York City–Newark were ±25% and unbiased compared to coincident Pandora measurements, ground-based total NO_2_ columns with relatively low uncertainties from AMFs that do not vary with NO_2_ vertical profile shape or surface albedo.^79^ During TRACER-AQ, GCAS NO_2_ columns were averaged to 250 m (cross-track) × 560 m (along track). GCAS flew onboard the NASA Johnson Space Center Gulfstream V (JSC GV) research aircraft on 11 days in September 2021. We use measurements from the 27 cloud-free flights sampling at least 60% of census tracts in the Houston MSA (Table S1). GCAS flew a repeated flight pattern in the morning (∼9–11:30 am LT), midday (∼11:30 am–2 pm LT), and afternoon (∼2:30–5 pm LT), sampling 83 ± 4% (±1σ) of tracts with similar, but not identical, demographics to the MSA (Tables S2–S3).

### Surface NO_2_*, O_3_, and Meteorological Measurements

NO_2_* observations are collected at 23 stations across the MSA (Figure S2a) and provided through the U.S. EPA Air Quality System.^80^ NO_2_* is mostly measured by decomposing NO_2_ to NO over a heated molybdenum catalyst and detecting NO by chemiluminescence, a technique with a known positive interference from other nitrogen compounds, which also thermally decompose across the catalyst at non-unity efficiency.^81−83^ The term NO_2_* acknowledges this interference, which, while affecting accuracy, has a smaller effect on precision.^84^ Two stations in the MSA are near-roadway monitors. We use O_3_ mixing ratios measured at 21 stations, many of which also house NO_2_* instruments (Figure S2b), converted to MDA8 O_3_. We use 1-h measurements of wind speed (resultant), wind direction, and air temperature and daily maximum temperatures collected at 23 stations (Figure S2c) with observations on at least 50% of days during O_3_ season, defined in Houston as March–November,^85^ when MDA8 O_3_ NAAQS exceedances are most likely to occur.

### Census Tract-Scale Inequalities

We calculate area-weighted mean NO_2_ TVCDs within 2020 census tract polygons across the Houston UA and MSA and population weight tract-average TVCDs using race and ethnicity data from the U.S. Census 5-year 2020 American Community Survey (ACS). The ACS subsamples census unit populations and applies a complex weighting process to account for variability in tract-level sampling rates and differential group response rates. The weighting process prioritizes accuracy over precision, which we manage using population-weighting and aggregation across the UA and MSA.^86,87^ Tract-scale NO_2_ inequalities with race-ethnicity are reported as relative (%) and absolute (molecules cm^–2^) differences between population-weighted NO_2_ TVCDs (eq S1^3,18,88^) for non-Hispanic/Latino Black and African Americans, Hispanics and Latinos of all races, and non-Hispanic/Latino Asians compared to non-Hispanic/Latino whites in tracts with populations equal to or greater than the mean across tracts with observations. NO_2_ differences with race and ethnicity are treated as a proxy for racism.

## Results and Discussion

### Evaluating Daily TROPOMI NO_2_ Inequalities in Houston, Texas

**Figure 2:**
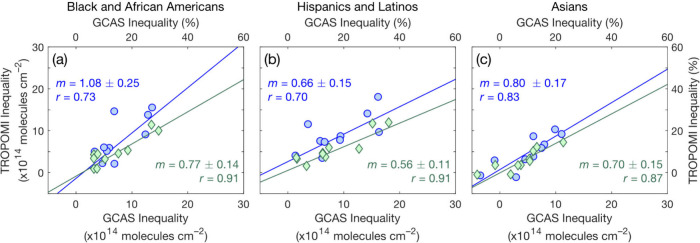
Spatiotemporally coincident (±30 min) relative (%) (blue circles) and absolute (molecules cm^–2^) (green diamonds) GCAS and TROPOMI NO_2_ inequalities during TRACER-AQ for Black and African Americans (a), Hispanics and Latinos (b), and Asians (c) in comparison to non-Hispanic/Latino whites with slopes (*m*), based on an unweighted bivariate linear regression, and Pearson correlation coefficients (*r*) of relative (blue) and absolute (green) inequalities.

We first compare spatially and temporally coincident daily census tract-scale TROPOMI NO_2_ inequalities against those computed using GCAS NO_2_ columns, which have sufficient spatial resolution to observe NO_2_ dispersion gradients. Correspondence between daily TROPOMI and GCAS inequalities is described using Pearson correlation coefficients and slopes derived from an unweighted bivariate linear regression of simultaneous observations, defined as occurring within ±30 min (Figure 2). TROPOMI and GCAS NO_2_ inequalities are strongly correlated, with *r* values of 0.70–0.83 (relative) and 0.87–0.91 (absolute), indicating daily TROPOMI NO_2_ TVCDs reflect the variability of spatially detailed GCAS observations day to day. Regression slopes are 0.66 ± 0.15 to 1.08 ± 0.25 for relative and 0.56 ± 0.11 to 0.77 ± 0.14 for absolute inequalities; therefore, daily TROPOMI NO_2_ TVCDs capture a major portion of tract-scale inequalities in Houston. Slopes for relative inequalities are larger than for absolute inequalities, with relative differences easier to distinguish using measurements coarser than distance decay gradients. This is consistent with results from daily observations in New York City–Newark^41^ and reinforces conclusions based on oversampled TVCDs in Houston by Demetillo et al.,^3^ where TROPOMI resolved comparable relative but lower absolute inequalities than GCAS during DISCOVER-AQ.

We test the sensitivity of daily TROPOMI census tract-scale NO_2_ inequalities to TROPOMI observation spatial resolution by comparing NO_2_ inequalities across the natural variability in daily mean TROPOMI pixel size, ranging 20–89 km^2^ with a mean of 39 ± 16 km^2^ (±1σ standard deviation) UA wide (May 2018–November 2022). Because daily inequalities are sensitive to observation coverage, we first remove days with NO_2_ observations in fewer than 20% of tracts in the domain (discussed below). We group observations according to thresholds defined by pixel-size quintiles, comparing mean inequalities for each threshold to those derived from the smallest 20% of pixels using 95% confidence intervals from bootstrapped distributions sampled with replacement 10^4^ times (Table S4). We do not observe statistically significant differences in mean daily TROPOMI inequalities outside of the 95% confidence intervals compared to the smallest pixels. The lack of pixel area dependence suggests most city-wide NO_2_ inequalities, and those that are observed by TROPOMI, are driven by spatially clustered NO_*x*_ sources. TROPOMI pixels are larger than the length scales of individual dispersion gradients; however, when NO_*x*_ sources are clustered into source regions, their gradients also spatially aggregate. TROPOMI resolves NO_2_ gradients on the scale of these source regions, if not individual sources, with the latter causing the information loss compared to GCAS.

Observed NO_2_ inequalities based on TVCDs are sensitive to the number of census tracts with NO_2_ measurements across the domain (UA or MSA).^41^ When observation coverage is low, inequalities tend to be based on TVCDs in census tracts less representative of city-wide demographics. In this case, census tracts where high numbers of residents are in population groups in the majority with respect to city area (not necessarily population count) are overrepresented in the calculation. The net effect is that population-weighted inequalities are based on census tracts that have higher populations of non-Hispanic whites than in the domain on average. In New York City–Newark, Dressel et al.^41^ found low observation coverage biased NO_2_ inequalities low by 6–7 percentage points and, as a result, identified minimum coverage threshold requirements for daily mean NO_2_ inequalities. We test sensitivity of mean daily TROPOMI NO_2_ inequalities in Houston, first applying a minimum coverage requirement of 20% of census tract with observations, then binning daily TVCDs by >20%, >40%, >60%, and >80% census tracts with observations. When bootstrap 95% confidence intervals (calculated with replacement 10^4^ times) for a lower coverage bin do not overlap with the 95% confidence interval for the >80% coverage bin, we identify a significant difference between inequalities. We select thresholds separately for each metric as the lowest coverage bin without a significant difference. Coverage thresholds range 20–40% for relative and absolute inequalities for each metric (Table S5) and are applied throughout. Mean daily TROPOMI NO_2_ inequalities in Houston exhibit less observational coverage sensitivity than in New York City–Newark.^41^

We compare mean daily NO_2_ inequalities to results based on NO_2_ TVCDs on the same subset of days oversampled to 0.01° × 0.01° (∼1 km × 1 km) using a physics-based algorithm^89^ prior to census tract averaging (Table 1). Oversampling averages measurements over time with large and overlapping pixels to a finer grid, allowing sub-pixel-scale spatial features to be recovered.^89^ The oversampling approach used here treats pixel-level observations as sensitivity distributions using a generalized two-dimensional super Gaussian spatial response function, appropriate for imaging grating spectrometers like TROPOMI. Relative mean daily and oversampled NO_2_ inequalities are equal to within associated uncertainties; however, absolute NO_2_ inequalities in mean daily TVCDs, which are already low relative to fine-scale airborne remote sensing (Figure 2), are as much as ∼30% higher than oversampled TVCDs. We see multiple possible explanations for this: oversampling is not enhancing spatial gradients relevant to describing census tract-scale NO_2_ inequality, which is instead determined by the spatial resolving power set by pixel size; there is limited NO_2_ variability on scales of 1–4 km as relevant to NO_2_ inequalities; and/or there is compensating information in the daily inequalities lost through time averaging.

**Table 1:** Mean Daily TROPOMI NO_2_ Inequalities at the MSA and UA Level (May 2018–November 2022) on Days Meeting Observation Coverage Thresholds, Inequalities Based on TROPOMI NO_2_ TVCDs Oversampled to 0.01° × 0.01°, 0.02° × 0.02°, 0.04° × 0.04°, and 0.06° × 0.06°, and Average Inequalities of the 15 TROPOMI Orbit Patterns That Cover the Houston UA Separately from Means and Oversampled TVCDs (0.01° × 0.01°)^a^

			Oversampled TROPOMI					Separately by TROPOMI Orbit	
	Mean Daily TROPOMI		MSA	UA				UA	
	MSA	UA	0.01° × 0.01°	0.01° × 0.01°	0.02° × 0.02°	0.04° × 0.04°	0.06° × 0.06°	Mean	Oversampled (0.01° × 0.01°)
	Relative Inequalities (%)								
Black and African Americans	17 ± 1	8 ± 1	18 ± 1	9 ± 1	9 ± 1	8 ± 1	8 ± 1	9 ± 1	9 ± 1
Hispanics and Latinos	23 ± 1	16 ± 1	25 ± 1	17 ± 1	17 ± 1	17 ± 1	16 ± 1	18 ± 1	16 ± 1
Asians	9 ± 1	–1 ± 1	11 ± 1	0 ± 1	1 ± 1	1 ± 1	2 ± 1	4 ± 1	2 ± 1
	Absolute Inequalities (×10^14^ molecules cm^–2^)								
Black and African Americans	6.4 ± 0.5	3.6 ± 0.3	5.0 ± 0.3	2.7 ± 0.3	2.7 ± 0.3	2.6 ± 0.3	2.6 ± 0.4	3.7 ± 0.5	2.8 ± 0.4
Hispanics and Latinos	8.8 ± 0.5	6.8 ± 0.4	7.2 ± 0.4	5.4 ± 0.3	5.4 ± 0.3	5.3 ± 0.3	5.2 ± 0.4	7.3 ± 0.5	5.3 ± 0.4
Asians	3.7 ± 0.4	0.3 ± 0.4	2.9 ± 0.3	0.1 ± 0.3	0.1 ± 0.3	0.4 ± 0.4	0.5 ± 0.4	0.2 ± 0.5	0.4 ± 0.4

a Uncertainties are expressed as standard mean errors.

**Figure 3:**
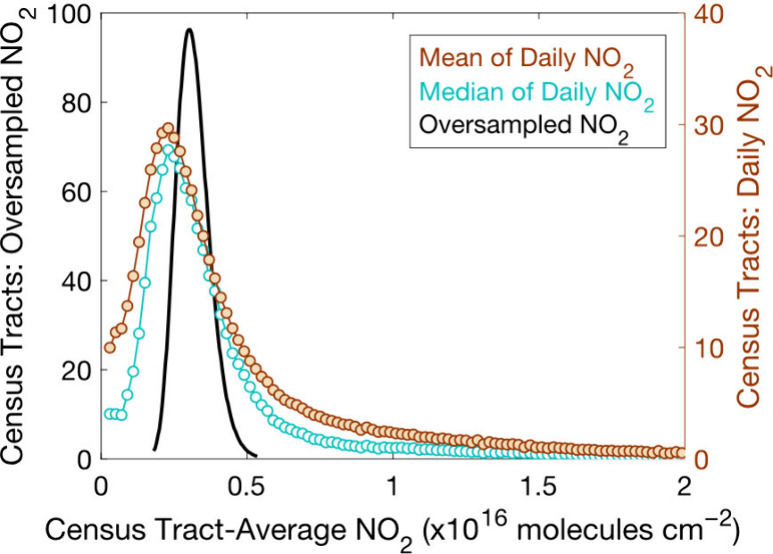
Lognormal distributions of census tract-average TROPOMI NO_2_ TVCDs in the Houston UA (May 2018–November 2022). Left axis: TVCDs oversampled to 0.01° × 0.01° (black line). Right axis: mean (brown filled circles) and median (cyan open circles) of distributions of daily observations.

First, we compare NO_2_ inequalities based on oversampled TVCDs over a range of grid sizes, finding no significant differences in relative or absolute inequalities when we oversample to 0.01° × 0.01°, 0.02° × 0.02°, 0.04° × 0.04° (the approximate TROPOMI nadir resolution), and 0.06° × 0.06°. In an analysis of 52 major U.S. UAs, Demetillo et al.^16^ also reported small differences in relative and absolute census tract-scale NO_2_ inequalities using TROPOMI TVCDs oversampled to 0.01° × 0.01° and 0.04° × 0.04°, with the exceptions of the narrow coastal Californian cities of Oakland, San Diego, and San Francisco, where NO_2_ inequalities based on TVCDs oversampled to 0.04° × 0.04° were biased low by 8–22% compared to TVCDs oversampled to 0.01° × 0.01°, suggesting oversampling enhances spatial gradients from coarser pixels when that variability exists.^16^ Second, we take advantage of the natural variability in TROPOMI pixel orientations, separately comparing NO_2_ inequalities based on oversampled TVCDs to mean NO_2_ TVCDs collected within individual S-5P orbits, thus eliminating the oversampling pixel overlap requirement. On average, for the 15 S-5P satellite orbits that fully cover the Houston UA, relative NO_2_ inequalities from oversampled and mean NO_2_ TVCDs are similar; however, absolute NO_2_ inequalities of mean TVCDs are ∼30% higher than oversampled TVCDs for Black and African Americans and Hispanics and Latinos (Table 1; Table S6), indicating the information loss is not simply because of time averaging, but smoothing during oversampling. In Figure 3, we compare mean and median distributions of tract-scale daily and oversampled (0.01° × 0.01°) TROPOMI TVCDs, fit assuming distributions are lognormal as is characteristic for NO_2_. Mean daily measurements span a wider range of NO_2_ conditions and retain more observations in the high tail of the distribution than oversampled TVCDs, with high NO_2_ values driving inequalities. Sun et al.^89^ report that oversampling, including with the physics-based algorithm used here, is more accurate when the grid is fine relative to a gradient with a smooth spatial response, for example, a city edge, while pixel means are more accurate for coarse grids and sharper spatial responses. Our results suggest absolute census tract-scale NO_2_ inequalities are more accurately represented using means, with TROPOMI pixels and typical oversampling grids being large relative to scale of dispersion. Research using oversampled NO_2_ TVCDs to identify NO_*x*_ point sources and infer NO_*x*_ emissions and NO_2_ lifetimes have improved absolute estimates by rotating spatially variable NO_2_ plumes to a common wind direction,^90−93^ an aspatial solution not applicable to describing census tract-scale NO_2_ inequalities, although potentially useful for informing related decision-making.

**Figure 4:**
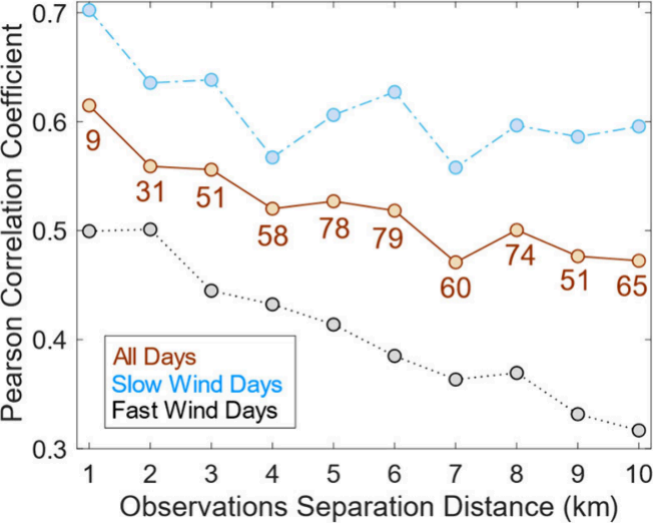
Median daily Pearson correlation coefficients between tract-averaged NO_2_ TVCDs and surface NO_2_* mixing ratios as a function of observation separation distance (km) on all days over May 2018–November 2022 (brown solid line) and on days in low (light blue dashed line) and high (black dotted line) quartile winds. We indicate the mean number of census tracts in the daily correlation at that distance each day, with similar statistics on low and high wind days.

To describe spatiotemporal variability in column-surface relationships, we compare daily tract-average TROPOMI TVCDs and daytime (12–3 pm LT) NO_2_* surface mixing ratios across the MSA as a function of their separation distance using Pearson correlation coefficients (*r*) over May 2018–November 2022 (Figure 4).^3,16,41,69^ We require NO_2_* mixing ratio data at four or more monitors in each 1-km distance bin per day and exclude near-roadway monitors, which are subject to hyperlocal effects. Surface NO_2_* and directly overhead TVCDs (defined as tract center points within 1 km of an NO_2_* monitor) are strongly correlated, with median *r* values of 0.62. Correlation coefficients decrease as the distance between observations increases, falling to 0.54 on average when tract-average TVCDs are 2–6 km from the nearest monitor and 0.48 at 7–10 km. This *r*-distance dependence indicates spatial variability in daily TROPOMI TVCDs follows NO_2_* patterns at the surface, with *r* decreases at 1–2 km consistent with length scales of NO_2_ dispersion gradients. If we consider uncertainties as standard mean errors based on the number of days with observations included in the daily average, uncertainties in *r* are typically ±0.01 and mean differences in *r* with distance are significant. However, column-surface relationships are variable daily, with standard deviations (1σ) of ∼0.3 in each distance bin. Daily correlation coefficients are lower than for oversampled TROPOMI TVCDs as reported in Demetillo et al.,^3^ especially at 1 km, meaning time averaging masks temporal variability in column-surface agreement. We also sort daily observations in the highest (>3.9 m s^–1^) and lowest (<2 m s^–1^) UA-wide mean daytime (12–3 pm LT) surface wind quartiles as a function of distance, as wind is a physical control over the inter-tract NO_2_ distribution. Daily column-surface correlations covary with wind speeds physically realistically, with stronger *r* values for slower winds and smaller *r* values with faster winds at all observation separation distances.

### Daily NO_2_ Inequalities

**Figure 5:**
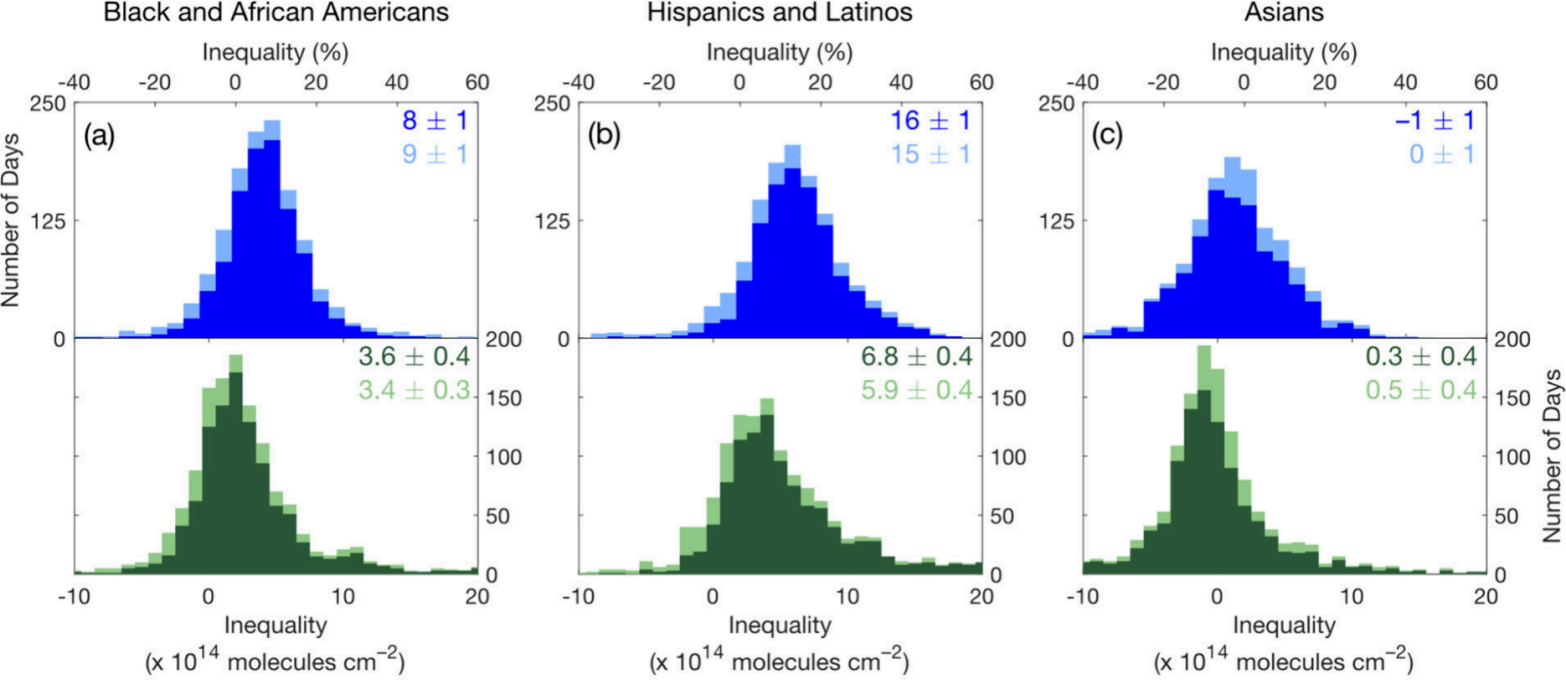
Daily UA-level TROPOMI NO_2_ inequalities (May 2018–November 2022). Relative (%) and absolute (molecules cm^–2^) inequalities on all days (light blue and light green, respectively) and on days meeting metric-specific coverage thresholds (bright blue and dark green, respectively) for Black and African Americans (a), Hispanics and Latinos (b), and Asians (c). Bootstrap mean inequalities, sampled with replacement 10^4^ times, are reported with uncertainties as 95% confidence intervals.

We calculate daily TROPOMI census tract-average NO_2_ inequalities over May 2018–September 2022 across the Houston UA and MSA (Table 1; Figure 5). Mean daily UA-level population-weighted NO_2_ TVCDs are 8 ± 1% and 16 ± 1% higher for Black and African Americans and Hispanics and Latinos compared to non-Hispanic/Latino whites, respectively. Neighborhoods near the Houston Ship Channel (Figure 1) with large populations of Black and African Americans and Hispanics and Latinos, e.g., Pasadena, Fifth Ward, Harrisburg/Manchester, and Galena Park, often have the highest NO_2_ concentrations. Mean population-weighted NO_2_ TVCDs for each group including non-Hispanic/Latino whites are shown in Table S7. Inequalities for Black and African Americans and Hispanics and Latinos increase to 17 ± 1% and 23 ± 1%, respectively, at the MSA level. Mean daily population-weighted NO_2_ TVCDs for Asians equal those for non-Hispanic/Latino whites within the UA but are 9 ± 1% higher across the MSA, mainly due to the inclusion of the large Asian population around Sugar Land in southwest Houston (Figure 1d). We observe larger inequalities at the MSA level, reflecting urban-suburban differences, compared to the UA, representing intraurban NO_2_ differences.^3,94^ UA and MSA-level relative (*r* = 0.83–0.92) and absolute (*r* = 0.88–0.95) inequalities are strongly correlated (Figure S3). Errors for mean inequalities are 95% confidence intervals, which we derive from bootstrapped distributions sampled with replacement 10^4^ times. Absolute census tract-scale NO_2_ inequalities are often lower than the precision of individual TROPOMI NO_2_ TVCDs, which have a median daily pixel-level precision of 9.9 × 10^14^ molecules cm^–2^ (approximately 30% of mean NO_2_ TVCDs) over May 2018–November 2022 in the Houston UA. However, this imprecision improves through spatial and temporal averaging,^95^ done here through population weighting over all census tracts in the UA or MSA and by reporting daily inequality results as means over many days. Sampling and nonsampling (e.g., measurement, coverage, nonresponse, and processing errors) errors in the ACS influence the accuracy and precision of tract-scale NO_2_ inequalities as well and, when random, also improve through averaging to higher geographic levels.

We report NO_2_ inequalities during 27 TRACER-AQ flights using GCAS separately in the late morning, midday, and afternoon (Table 2). Relative inequalities are not statistically significantly different with time of daytime, although there may be a tendency toward lower relative inequalities at midday. Absolute NO_2_ inequalities are significantly higher in the morning than midday and afternoon, and there are multiple factors that could influence these differences. While wind speeds are similar on average during all flights, the atmosphere is typically more stable in morning than at midday, affecting the NO_2_ distribution in the nearfield of NO_*x*_ sources,^19^ with convective mixing common in the afternoon in Houston. The surface mixed layer height is typically shallower in the morning than afternoon; however, this will have a larger effect on surface concentrations than TVCDs. We also expect higher rush hour NO_*x*_ emissions and longer NO_2_ chemical lifetimes^96^ in the morning and late afternoon compared to midday. Diurnal variability in absolute inequalities has implications for interpreting observations from TROPOMI, which collects measurements at 12–3 pm LT over Houston, and the recently-launched TEMPO (Tropospheric Emissions: Monitoring of Pollution) instrument, which scans North America hourly during daylight hours from onboard a geostationary satellite.^97^ Our analysis in the New York City–Newark UA found fewer statistically significant morning-afternoon differences in absolute NO_2_ inequalities,^41^ suggesting there is more to learn from TEMPO concerning temporal variability in the NO_2_ distribution. Because GCAS subsampled the MSA, we also report mean daily TROPOMI NO_2_ inequalities (May 2018–November 2022) along a representative TRACER-AQ flight for comparison (Table 2).

**Table 2:** Relative and Absolute Mean GCAS NO_2_ Inequalities in the Houston MSA During TRACER-AQ in the Morning (9–11:30 am LT), at Midday (11:30 am–2 pm LT), and in the Afternoon (2:30–5 pm LT); Relative and Absolute Mean Daily TROPOMI NO_2_ Inequalities (May 2018–November 2022) along a Representative TRACER-AQ Flight Raster (Afternoon, 25 September 2021); and GCAS Inequalities along Spatially Coincident TRACER-AQ and DISCOVER-AQ Tracts during TRACER-AQ (2021) and DISCOVER-AQ (2013)^a^

	GCAS TRACER-AQ morning	GCAS TRACER-AQ midday	GCAS TRACER-AQ afternoon	TROPOMI along TRACER-AQ raster	2021 GCAS (TRACER-AQ)	2013 GCAS (DISCOVER-AQ)
	Relative Inequalities (%)					
Black and African Americans	17 ± 7	12 ± 6	13 ± 4	13 ± 1	9 ± 8	10 ± 6
Hispanics and Latinos	27 ± 4	20 ± 6	25 ± 2	22 ± 1	24 ± 6	20 ± 5
Asians	12 ± 10	13 ± 10	11 ± 4	3 ± 2	9 ± 6	11 ± 4
	Absolute Inequalities (×10^14^ molecules cm^–2^)					
Black and African Americans	14.4 ± 5.8	6.8 ± 3.9	8.2 ± 2.6	6.0 ± 0.7	4.9 ± 4.6	10.9 ± 6.3
Hispanics and Latinos	22.7 ± 4.4	11.9 ± 3.4	16.0 ± 3.6	10.6 ± 1.0	16.4 ± 3.7	19.3 ± 5.9
Asians	17.0 ± 12.9	9.2 ± 6.9	7.6 ± 3.8	2.5 ± 1.0	9.6 ± 6.2	9.4 ± 3.7

a Airborne and TROPOMI uncertainties are 95% confidence intervals of bootstrap mean inequalities, sampled with replacement 10^4^ times.

GCAS NO_2_ measurements in Houston collected during TRACER-AQ and DISCOVER-AQ offer observational insight into trends from 2013 to 2021 (Table 2). We compare weekday population-weighted, tract-average NO_2_ columns in spatially coincident census tracts along representative TRACER-AQ and DISCOVER-AQ flight patterns (SI Appendix 1; Figure S4; Tables S8–S11). We calculate inequalities using the 2020 ACS for both DISCOVER-AQ and TRACER-AQ to allow comparisons across the same tracts and isolate effects of changes in NO_2_ concentrations from demographics. We find relative NO_2_ inequalities are statistically indistinguishable, with overlapping 95% confidence intervals for NO_2_ inequalities in 2013 and 2021 and by the Wilcoxon rank sum test, a non-parametric two-sample t-test. While absolute inequalities were always lower during TRACER-AQ than DISCOVER-AQ, they were variable day to day, in addition to the relatively small number of aircraft observations, such that we lack the precision on their means (not the observations themselves) to interpret the differences. UA-wide mean NO_2_* mixing ratios were slightly higher and more variable during DISCOVER-AQ (6.7 ± 6.2 ppb) than TRACER-AQ flights (6.0 ± 4.3 ppb); winds were slower during TRACER-AQ (2.1 ± 0.8 m s^–1^) than DISCOVER-AQ (3.1 ± 1.2 m s^–1^). Slower mean winds during TRACER-AQ may have worsened inequalities, while lower NO_2_* corresponds to lower absolute inequalities (discussed below). Previous work has shown downward NO_*x*_ emissions trends have not reduced relative NO_2_ inequalities in U.S. cities using NO_2_ empirical models;^12,88^ however, this has not yet been demonstrated with observations directly to our knowledge.

Relationships between daily UA-level census tract-scale TROPOMI NO_2_ inequalities, surface winds, and overall NO_2_ pollution (Table 3; Figures S5–S7) underscore the need for locally targeted controls over sector-based approaches to reducing NO_2_ disparities. Absolute NO_2_ inequalities are moderately negatively associated with wind speeds for most groups, as faster winds distribute NO_2_ away from NO_*x*_ sources, showing NO_2_ inequalities arise from the distribution of NO_*x*_ sources, as well as that daily NO_2_ inequalities vary meaningfully with relevant atmospheric conditions. Absolute NO_2_ inequalities moderately correlate with UA-mean surface NO_2_* and NO_2_ TVCDs in the winter and during O_3_ season for most metrics. At the same time, relative inequalities are more-weakly associated with overall NO_2_. Differences in these correlations for absolute and relative NO_2_ inequalities manifest from NO_*x*_ sources being systematically located in Black and African American and Hispanic and Latino, as NO_2_ concentrations in the nearfield of emitters are more temporally variable than the physical locations of NO_*x*_ sources. As a consequence, emissions reductions that maintain unequal source distributions, such as sector-based approaches, lower overall NO_2_ pollution and absolute differences between groups but have little effect on relative inequalities, which require location-specific policy interventions.^98^

**Table 3:** Spearman Rank Correlation Coefficients (2018–2022) with *p* < 0.050 in Winter and O_3_ Season: Daily Absolute TROPOMI Inequalities and Daytime (12–3 pm LT) Surface Wind Speed, NO_2_* Mixing Ratios, and Daily UA-Level TROPOMI NO_2_ TVCDs and Daily Relative TROPOMI Inequalities and Daytime NO_2_* Mixing Ratios and UA-Level TROPOMI NO_2_ TVCDs

	Absolute Inequality Correlations			Relative Inequality Correlations	
	Wind Speed	Surface NO_2_*	NO_2_ TVCDs	Surface NO_2_*	NO_2_ TVCDs
	Winter (December–February)				
Black and African Americans	–0.40	0.44	0.55	0.25	0.26
Hispanics and Latinos	–0.62	0.67	0.67	0.31	0.17
Asians			0.21		0.21
	O_3_ Season (March–November)				
Black and African Americans	–0.34	0.48	0.65	0.17	0.20
Hispanics and Latinos	–0.51	0.61	0.77	0.24	0.26
Asians	–0.17		0.15		0.07

### NO_2_ Inequalities and O_3_ Air Quality

**Figure 6:**
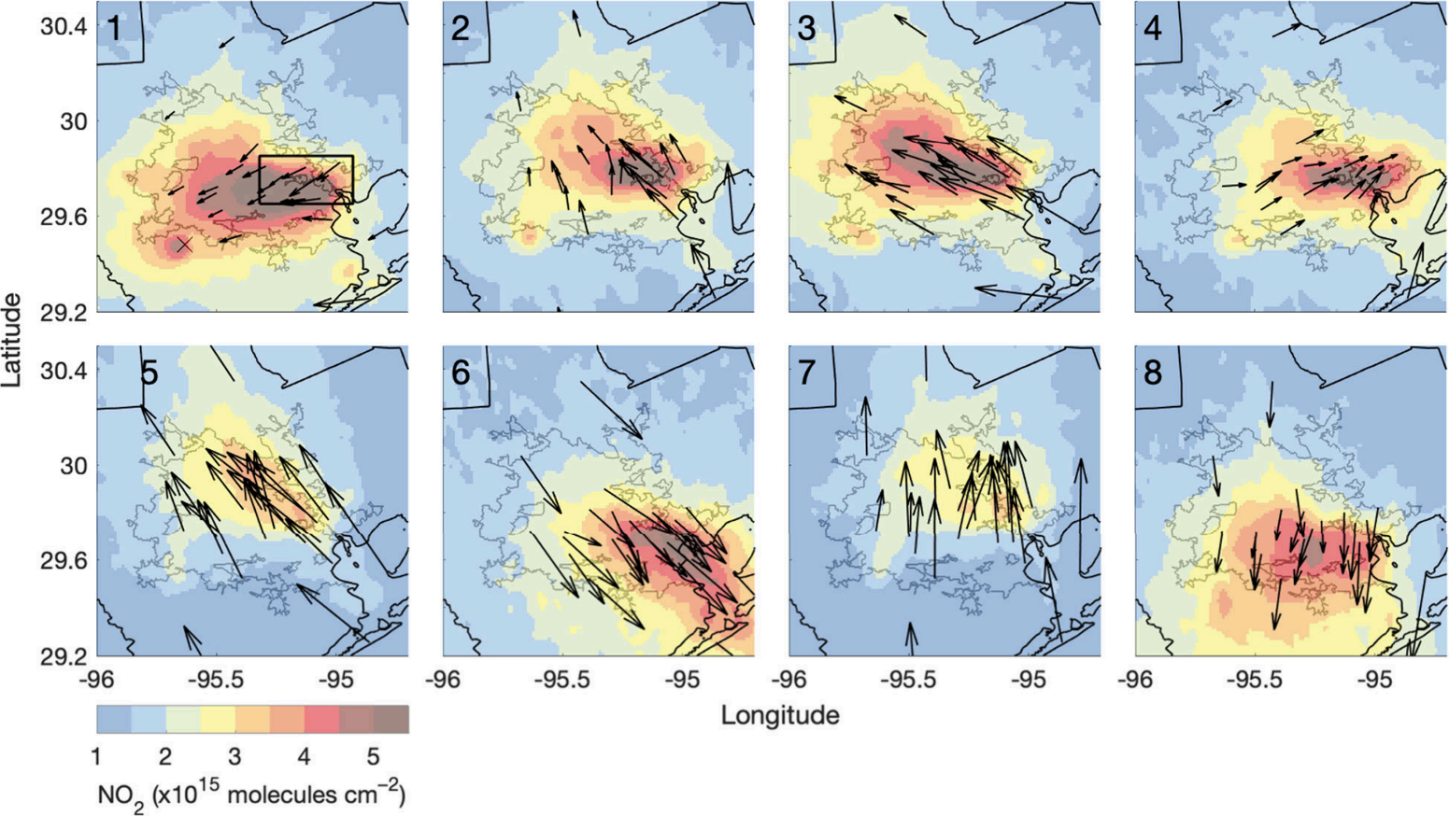
Distinct mean daytime (12–3 pm LT) wind clusters during O_3_ season (March–November) over May 2018–November 2022 in the Houston MSA and corresponding TROPOMI NO_2_ TVCDs oversampled to 0.01° × 0.01°. Wind vector length is proportional to wind speed, with mean wind speeds given in Table 4. The W.A. Parrish Generating Station is indicated with an × and the Houston Ship Channel with a thick black box in cluster 1. The thin inner gray and outer black lines are the UA and MSA boundaries, respectively.

We use daily observations of NO_2_ inequalities to investigate relationships between neighborhood-level NO_2_ distributions and O_3_ air quality. First, applying an established approach to understanding the influence of meteorology on O_3_ variability in Houston, we disaggregate observations by winds using principal component and cluster analysis,^48,53,99−103^ presenting cluster characteristics that include census tract-scale NO_2_ inequalities. We generate one two-dimensional principal component for mean daytime (12–3 pm LT) u and v resultant winds during O_3_ season, which captures 88% of the observed variability in u and v components. We then apply *k*-means clustering with 1,000 iterations to generate eight wind clusters, with the first centroid selected at random, from the iteration with the lowest total sum of distances (Figure 6; Table 4). We selected the optimal number of clusters, allowed to range 1–10, using the Calinski-Harabasz criterion, maximizing the ratio of the between-cluster variance to the within-cluster variance with respect to the number of clusters.^104^ We confirmed the identified number of clusters using the elbow method with 10^3^ iterations, with the optimal number of clusters based on the variance explained.^105^ Eight clusters balanced clarity and complexity relevant to relationships between NO_2_ inequalities and MDA8 O_3_. Missing daytime winds are filled using measurements from the closest proximity monitor with observations. We renamed the clusters 1–8 from most to least frequent MDA8 O_3_ NAAQS exceedances. The analysis reproduces results in the literature, with high O_3_ days associated with easterly and east-southeasterly winds.^48,53,100,103^Figure 6 highlights the variability in NO_2_ spatial distributions lost through averaging (Figure 1c), with results based on long-term or annual averages representing conditions that infrequently occur.

**Table 4:** Mean Daytime Wind Cluster Characteristics: Number of Days in Each Cluster; MSA-Mean Wind Speed (±1σ) and Direction; MSA-Level MDA8 O_3_ NAAQS Exceedances, Both Number and Frequency; UA-Mean NO_2_* (±1σ); and Mean Daily TROPOMI Relative and Absolute Inequalities Based on Bootstrapped Distributions Sampled with Replacement 10^4^ Times with Uncertainties as 95% Confidence Intervals

	Cluster							
	1	2	3	4	5	6	7	8
Number of days	186	279	193	112	170	48	162	136
Wind speed (m s^–1^)	0.7	1.2	2.2	1.3	3.7	4.2	3.5	2.5
Wind direction	easterly	southerly	east southeasterly	westerly	southeasterly	northwesterly	southerly	northwesterly
Temperature (°C)	28	32	27	30	28	23	30	23
O_3_ NAAQS exceedances	49	38	22	12	9	2	5	4
O_3_ exceedance frequency (%)	26	14	11	11	5	4	3	3
NO_2_* (ppb)	7.4	6.6	7.2	6.1	5.1	5.5	3.9	6.7
	UA							
	Mean Daily Relative Inequalities (%)							
Black and African Americans	11 ± 1	13 ± 1	10 ± 2	9 ± 3	9 ± 2	–3 ± 3	10 ± 1	6 ± 2
Hispanics and Latinos	19 ± 2	22 ± 2	17 ± 2	18 ± 6	14 ± 2	14 ± 3	13 ± 2	9 ± 2
Asians	9 ± 2	0 ± 1	–1 ± 2	–6 ± 2	–3 ± 2	–15 ± 4	–0 ± 1	4 ± 2
	Mean Daily Absolute Inequalities (×10^14^ molecules cm^–2^)							
Black and African Americans	5.9 ± 1.1	5.0 ± 0.8	3.7 ± 0.6	3.3 ± 0.9	2.3 ± 0.6	–0.7 ± 0.5	2.1 ± 0.3	2.1 ± 0.5
Hispanics and Latinos	9.5 ± 1.4	9.1 ± 1.4	6.9 ± 0.8	8.1 ± 1.5	3.7 ± 0.6	4.1 ± 0.8	2.9 ± 0.4	2.9 ± 0.5
Asians	5.3 ± 1.5	–0.1 ± 0.5	–0.4 ± 1.0	–0.9 ± 1.4	–0.7 ± 0.4	–3.1 ± 0.5	–0.0 ± 0.3	1.8 ± 0.7

MDA8 O_3_ NAAQS exceedances are most frequent in cluster 1, when winds are on average slow and easterly—corresponding to the largest absolute daily TROPOMI race-ethnicity inequalities (Table 4). Cluster 1 is the primary wind condition in which we observe statistically significant UA-level inequalities for Asians, with NO_2_ from the Ship Channel transported toward Sugar Land in southwest Houston and stagnant NO_*x*_ emissions around the nearby coal-fired W.A. Parrish Generating Station. This explains why NO_2_ inequalities for Asians are not strongly correlated with wind speed or overall NO_2_ pollution level (Table 3). MDA8 O_3_ NAAQS exceedances are also common in clusters 2–4, when winds are slow (∼1.6 m s^–1^) and east-southeasterly, southerly, and westerly, with elevated UA-level absolute daily TROPOMI NO_2_ inequalities for all groups except Asians. Clusters 5–8 include the fewest number of O_3_ NAAQS exceedances, occurring on <5% of days. These clusters are characterized by faster winds, lower UA-mean NO_2_*, and lower absolute tract-scale daily TROPOMI NO_2_ inequalities. Wind conditions have less influence on relative NO_2_ inequalities, as winds do not affect the locations of NO_*x*_ sources. Observed correspondence between MDA8 O_3_ and absolute census tract-scale NO_2_ inequalities indicates similar atmospheric conditions exacerbate both phenomena and/or high O_3_ and NO_2_ inequalities are linked chemically.

**Figure 7:**
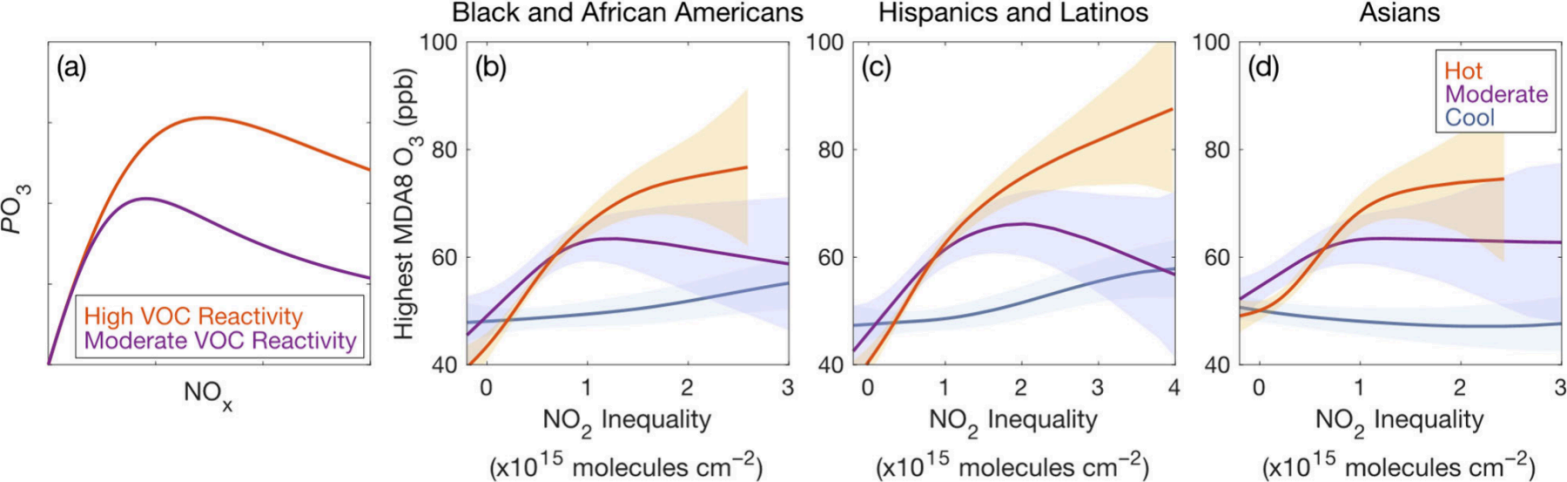
Analytical model demonstrating relationships between *P*O_3_, NO_*x*_, and VOC-OH reactivity (a). GAMs of daily MSA-level absolute TROPOMI NO_2_ inequalities (molecules cm^–2^) versus highest daily MDA8 O_3_ (ppb) during O_3_-season (March–November 2018–2022) on days meeting coverage thresholds under moderate (purple) and high (orange) daily maximum temperatures for Black and African Americans (b), Hispanics and Latinos (c), and Asians (d). Envelopes are 95% confidence intervals.

*P*O_3_ varies nonlinearly with NO_2_ concentrations (Figure 7a); therefore, NO_2_ inequalities and city-wide O_3_ air quality are potentially coupled chemically. Briefly, *P*O_3_ increases with increasing NO_*x*_ when NO is the limiting reagent in O_3_-forming radical cycling (*P*O_3_ chemistry is NO_*x*_ limited). *P*O_3_ decreases with increasing NO_*x*_ when NO_2_ predominately combines with OH to produce nitric acid, reducing O_3_-forming reactions between OH and VOCs (*P*O_3_ is NO_*x*_ suppressed). This nonlinear chemistry has important regulatory consequences, as NO_*x*_ decreases improve O_3_ air quality when chemistry is NO_*x*_ limited, while the same reductions worsen NO_*x*_-suppressed O_3_. When *P*O_3_ dominates the O_3_ mass balance, MDA8 O_3_ varies as the integral of *P*O_3_ across the intraurban NO_2_ heterogeneity, and, in Houston, NO_*x*_-limited and suppressed conditions are both present.^56^ Because *P*O_3_ depends nonlinearly on NO_2_, we describe O_3_-season relationships between NO_2_ inequalities and the highest daily MSA-level MDA8 O_3_ using a generalized additive model (GAM), a regression approach previously applied to nonlinear systems, including O_3_.^106−110^*P*O_3_ also depends nonlinearly on VOC reactivity to OH, defined as the sum of the product of VOC concentrations and their bimolecular reaction rate with OH.^111^ Temperature is a proxy for VOC-OH reactivity where a major portion of VOC emissions are temperature dependent, verifiable through the observed O_3_-NO_2_ dependence under different temperatures.^112^ To consider VOC-OH reactivity, we apply the GAM separately under low (<25°C), moderate (25–28°C), and high (>28°C) daytime mean temperatures conditions. Results informing GAM selection and evaluation are available in the Supporting Information (SI Appendix 2; Tables S12–13; Figures S8–S16).

GAMs of MDA8 O_3_ versus NO_2_ inequalities reproduce the nonlinear dependence of *P*O_3_ on NO_2_ concentrations (Figure 7). The highest MDA8 O_3_ occur hot days, i.e., under higher VOC-OH reactivity conditions, and when absolute NO_2_ inequalities are large. We observe lower MDA8 O_3_ when temperatures are moderate (lower VOC-OH reactivity) and NO_2_ inequalities are large, with similar MDA8 O_3_ to hot days when NO_2_ is more evenly distributed (*P*O_3_ is NO_*x*_ limited). At low temperatures, relationships between MDA8 O_3_ and NO_2_ inequalities suggest a more limited role for *P*O_3_ on MDA8 O_3_. A key observation is that the transition between NO_*x*_-limited and NO_*x*_-suppressed *P*O_3_ chemistry that is near peak MDA8 O_3_ occurs at higher absolute NO_2_ inequalities under higher temperature conditions, consistent with hotter temperatures corresponding to higher VOC-OH reactivities, which in turn require more NO_2_ to drive nitric acid production.^112^ While at very high NO concentrations O_3_ can be titrated to NO_2_, O_3_ titration does not have the same functional form as *P*O_3_ with VOC-OH reactivity versus NO_2_.

The GAMs demonstrate that NO_2_ inequalities affect *P*O_3_ chemistry and not merely that MDA8 O_3_ and NO_2_ inequalities covary under certain atmospheric conditions. We note, it is not the inequalities per se, but the unequal NO_2_ distributions resulting from NO_*x*_ sources being disproportionately located in a subset of neighborhoods that drives *P*O_3_. That said, NO_*x*_ emission sources overburden communities of color because of environmental racism in historical and contemporary decision-making. Past research has already shown that *P*O_3_ chemistry is spatially heterogenous within Houston,^46,49,56,113,114^ and, because *P*O_3_ chemistry is nonlinear, it follows logically that the same NO_*x*_ emission reductions applied evenly across a city would be less effective than a series of localized controls responsive to specific *P*O_3_ mechanisms (NO_*x*_ limited versus NO_*x*_ suppressed) as they vary in space. Wang et al.^58^ used the adjoint of the Community Multiscale Air Quality model focused on California to determine that *P*O_3_ is disproportionately sensitive to spatially localized controls. Our work implies that NO_*x*_ emissions controls that eliminate neighborhood-level NO_2_ inequalities will have O_3_ air quality co-benefits, with regulatory decision-making consolidating NO_*x*_ sources in a subset of Houston neighborhoods hindering O_3_ NAAQS compliance. While MDA8 O_3_ is largely NO_*x*_ limited with respect to NO_2_ inequalities on high temperature days, MDA8 O_3_ is more NO_*x*_ suppressed as a function of NO_2_ inequalities when temperatures are moderate, meaning even steeper NO_*x*_ reductions that also have the effect of decreasing NO_2_ inequalities are required to lower O_3_ under these conditions. Based on observed differences in correlations between absolute and relative NO_2_ inequalities with overall NO_2_ (Table 3), decreases in NO_2_ inequalities, and hence MDA8 O_3_, require locally targeted NO_*x*_ reductions in neighborhoods where residents are primarily Black, Latinx, and Asian.

### Implications

In Houston, daily TROPOMI NO_2_ TVCDs capture a major portion of census tract-scale NO_2_ inequalities compared to spatiotemporally coincident GCAS measurements that resolve length scales of dispersion. Mean daily TROPOMI NO_2_ inequalities are insensitive to TROPOMI pixel size after the initial information loss with respect to GCAS. In Houston, and other U.S. cities, communities of color are statistically overburdened by air pollution sources,^98,115,116^ including NO_*x*_ sources.^16^ This is a consequence of historical (e.g., redlining) and contemporary (e.g., permitting) decision-making that clusters emission sources in a subset of city neighborhoods, creating source regions such as the Houston Ship Channel, in combination with historical and contemporary policies and practices causing and reinforcing housing segregation,^1^ including white violence, housing discrimination, and separating communities with freeways.^117−121^ When NO_*x*_ sources are in close proximity, their individual pollutant decay gradients also spatially aggregate; as a result, a major portion of inequalities persist over spatial scales greater than length scales of dispersion, the physical process motivating the application of very-high spatial resolution models and measurements. Fine-scale observations are therefore not always required as evidence of air pollution inequalities or to inform related policy making and accountability. While daily TVCDs are coarse (20–89 km^2^), they retain a wider range of NO_2_ values, especially in the high tail of the NO_2_ distribution, which drive inequalities. Daily mean NO_2_ TVCDs result in higher, and therefore more accurate, absolute NO_2_ inequalities than oversampled TVCDs (0.01° × 0.01°), as TROPOMI pixels and oversampling grids are large relative to the scale of dispersion. This has relevance to future work based on TEMPO observations, which are not anticipated to meet the pixel overlap requirements for oversampling.

We find that neighborhood-level NO_2_ inequalities and city-wide O_3_ are coupled air quality issues in Houston. GAMs relating NO_2_ inequalities and MDA8 O_3_ under different temperature conditions reproduce established nonlinear relationships between *P*O_3_, NO_2_, and VOC-OH reactivity. This has policy consequences, producing empirical evidence that MDA8 O_3_ is sensitive to the spatial distribution of NO_*x*_ emissions reductions. O_3_ control is typically approached through sector-based NO_*x*_ and VOC emissions reductions without also considering distributive inequalities in O_3_ precursors.^122^ However, we find that targeted NO_*x*_ emissions reductions where NO_*x*_ sources are clustered—in communities of color—would lower both NO_2_ inequalities and city-wide MDA8 O_3_ in Houston, especially on hot days when MDA8 O_3_ is highest. This means that permitting and other policies concentrating sources in a subset of Houston neighborhoods affect O_3_ NAAQS attainment and calls for a reconceptualization of decision-making to include facility/emissions location.

While there is growing evidence that locally-targeted regulatory interventions are required to reduce and eliminate air pollution disparities,^41,98^ there are barriers to their adoption, as community-focused air quality plans and recommendations potentially cannot be pursued through policy making at any level.^123^ Houston and Pasadena (which is in the Houston UA) are among the few major U.S. municipalities without formal zoning, an established tool for localities to influence their own land use, including air pollution source distribution, through the institution of bans, programs, and environmental review processes.^124^ Additionally, Houston’s efforts to address air quality concerns through the local ordinance process have been invalidated by the Texas Supreme Court,^125,126^ further limiting the city from regulating emissions from facilities permitted by the Texas Commission on Environmental Quality (TCEQ). TCEQ does not have an office or staff focused on environmental justice, chooses not to use that term (any relevant activities are instead described as Title VI compliance), and continues to issue permits without considering cumulative impacts, including facility clustering. However, TCEQ does have a commitment to O_3_ compliance,^127^ making this a politically available pathway for addressing inequality in absence of other approaches. Here, we demonstrate that MDA8 O_3_ varies as a function of these neighborhood-level NO_2_ inequalities, with locally-targeted NO_*x*_ emissions controls required to address NO_2_ disparities and having substantial O_3_ air quality co-benefits. This conclusion has policy relevance as the state has the authority, resources, and initiative to meet the O_3_ NAAQS and is also evidence that TCEQ must contend with practices and policies of environmental racism to improve O_3_ air quality.

## Supporting Information Available

Surface wind roses corresponding to Figure 1, surface monitor locations, detailed TRACER-AQ inequality results, population weighting equation, TROPOMI inequalities as a function of observation coverage and pixel area, comparison of oversampled and time-averaged inequalities by S-5P orbit, mean daily TROPOMI population-weighted NO_2_, correlations between daily UA and MSA-level inequalities, details for the comparison between DISCOVER-AQ and TRACER-AQ inequalities, scatterplots of NO_2_ inequalities versus surface winds and NO_2_*, and technical details on generalized additive model (GAM) construction, including comparisons of GAM methods (PDF)
