# Supplementary material for: Neighborhood-Level Nitrogen Dioxide Inequalities Contribute to Surface Ozone Variability in Houston, Texas
Source: ACS EST Air. 2024 Jul 30;1(9):973–88. doi: 10.1021/acsestair.4c00009 (PMC11406531; doi:10.1021/acsestair.4c00009)
Supplement: Supplementary file 1 — ea4c00009_si_001.pdf [file ea4c00009_si_001.pdf]

Supporting Information

Neighborhood-level nitrogen dioxide inequalities contribute to surface ozone variability in Houston, Texas

AUTHOR NAMES: Isabella M. Dressel[1], Sixuan Zhang[1], Mary Angelique G. Demetillo[1,2], Shan Yu[3], Kimberly Fields[4], Laura M. Judd[2], Caroline R. Nowlan,[5] Kang Sun[6,7], Alexander Kotsakis[8], Alexander J. Turner[9], and Sally E. Pusede[1]*

*Corresponding author: sepusede@virginia.edu

AUTHOR ADDRESSES:

[1]Department of Environmental Sciences, University of Virginia, Charlottesville, Virginia 22904, United States

[2]NASA Langley Research Center, Hampton, Virginia 23681, United States

[3]Department of Statistics, University of Virginia, Charlottesville, Virginia 22904, United States

[4]Carter G. Woodson Institute for African American and African Studies, University of Virginia, Charlottesville, Virginia 22904, United States

[5]Atomic and Molecular Physics Division, Harvard Smithsonian Center for Astrophysics, Cambridge, Massachusetts 02138, United States

[6]Department of Civil, Structural and Environmental Engineering, University at Buffalo, Buffalo, New York 14260, United States

[7]Research and Education in eNergy, Environment and Water (RENEW) Institute, University at Buffalo, Buffalo, New York 14260, United States

[8]NASA Goddard Space Flight Center, Greenbelt, Maryland 20771, United States

[9]Department of Atmospheric Sciences, University of Washington, Seattle, Washington 98195, United States

SI includes: 36 pages, 16 figures, 13 tables, and 1 equation

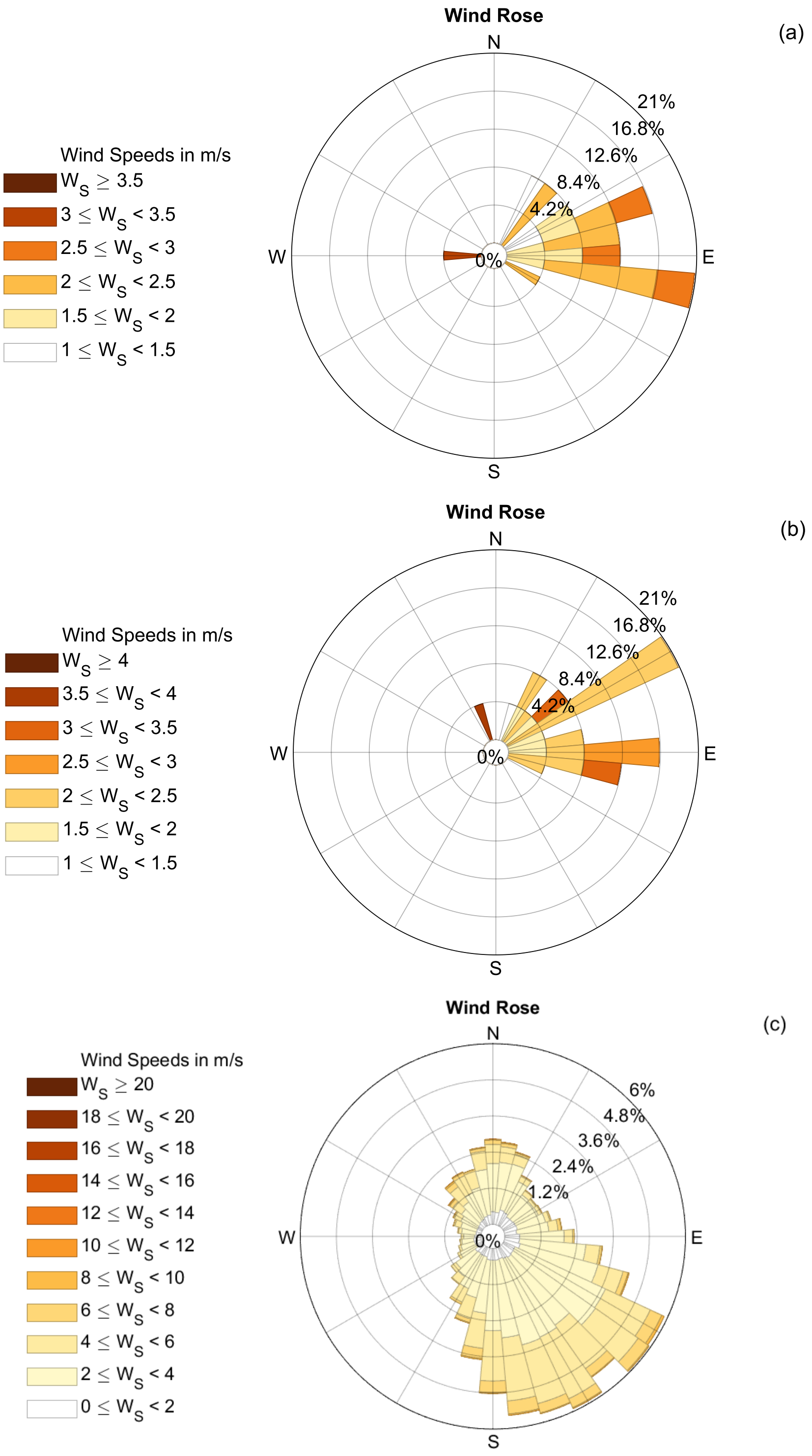

Figure S1. Wind roses in the mid-afternoon (2–5 pm) on 25 September 2021 (a), early afternoon (12–3 pm) on 25 September 2021 (b), and early afternoon (12–3 pm) from May 2018–November 2022 (c).[1]

Table S1. GCAS NO_2 inequalities during TRACER-AQ along rasters with >60% coverage of Houston MSA census tracts.

		Relative Inequality (%)			Absolute Inequality (x10^{14} molecules cm^{-2})		
Date	Raster	Black and African Americans	Hispanics and Latinos	Asians	Black and African Americans	Hispanics and Latinos	Asians
9/1/2021	1	30 ± 4	36 ± 3	–6 ± 4	12.8 ± 1.6	16.5 ± 1.5	–2.2 ± 1.3
9/1/2021	2	24 ± 3	34 ± 3	–4 ± 3	11.4 ± 1.5	16.9 ± 1.5	–1.5 ± 1.2
9/3/2021	1	28 ± 3	34 ± 3	2 ± 3	16.1 ± 1.8	20.2 ± 1.7	1.1 ± 1.5
9/3/2021	2	13 ± 3	28 ± 3	–3 ± 3	5.2 ± 1.1	12.2 ± 1.1	–1.0 ± 0.9
9/8/2021	1	2 ± 3	23 ± 3	–6 ± 3	1.2 ± 2.3	20.2 ± 2.5	–4.8 ± 2.4
9/8/2021	2	8 ± 3	19 ± 3	–4 ± 3	4.5 ± 1.6	12.0 ± 1.7	–2.4 ± 1.6
9/8/2021	3	5 ± 2	21 ± 2	1 ± 3	3.0 ± 1.5	13.1 ± 1.5	0.3 ± 1.5
9/9/2021	1	–4 ± 3	23 ± 3	–15 ± 3	–2.5 ± 1.8	16.6 ± 2.0	–8.6 ± 1.9
9/9/2021	2	10 ± 3	7 ± 3	15 ± 3	5.9 ± 2.0	4.0 ± 1.8	9.2 ± 2.1
9/9/2021	3	10 ± 2	24 ± 2	9 ± 2	4.5 ± 1.0	11.4 ± 1.0	4.0 ± 0.9
9/10/2021	1	24 ± 2	28 ± 3	25 ± 2	18.6 ± 1.9	21.7 ± 2.0	19.0 ± 1.9
9/10/2021	2	28 ± 3	26 ± 3	45 ± 3	18.3 ± 2.2	16.5 ± 2.0	32.0 ± 2.4
9/10/2021	3	15 ± 2	26 ± 2	13 ± 2	7.6 ± 1.0	14.2 ± 1.1	6.7 ± 0.9
9/11/2021	1	27 ± 2	27 ± 2	23 ± 2	14.9 ± 1.4	15.1 ± 1.4	12.7 ± 1.3
9/11/2021	2	10 ± 3	11 ± 3	20 ± 3	4.7 ± 1.4	5.4 ± 1.4	9.7 ± 1.6
9/23/2021	1	18 ± 3	25 ± 3	29 ± 3	21.0 ± 3.5	31.3 ± 3.6	37.2 ± 3.9
9/23/2021	2	14 ± 3	20 ± 3	23 ± 3	11.9 ± 2.3	17.1 ± 2.3	19.8 ± 2.4
9/23/2021	3	17 ± 3	27 ± 3	19 ± 3	14.5 ± 2.3	24.5 ± 2.3	16.8 ± 2.4
9/24/2021	1	15 ± 4	15 ± 3	40 ± 3	19.5 ± 4.7	18.7 ± 4.5	59.3 ± 5.1
9/24/2021	2	–7 ± 3	9 ± 3	–5 ± 3	–5.4 ± 2.3	7.4 ± 2.4	–3.6 ± 2.6
9/24/2021	3	11 ± 3	23 ± 3	10 ± 3	8.2 ± 2.2	18.6 ± 2.2	7.5 ± 2.4
9/25/2021	1	28 ± 3	31 ± 3	14 ± 4	32.0 ± 3.7	36.8 ± 3.5	15.1 ± 4.0
9/25/2021	2	21 ± 2	33 ± 2	18 ± 2	12.0 ± 1.3	20.6 ± 1.3	10.5 ± 1.1
9/25/2021	3	17 ± 2	27 ± 2	18 ± 2	8.2 ± 1.0	13.9 ± 1.1	8.7 ± 1.0
9/26/2021	1	20 ± 2	26 ± 2	14 ± 2	13.3 ± 1.4	17.6 ± 1.5	8.8 ± 1.5
9/26/2021	2	5 ± 3	16 ± 3	8 ± 3	2.0 ± 1.1	6.9 ± 1.1	3.1 ± 1.2
9/26/2021	3	21 ± 2	27 ± 2	8 ± 2	7.3 ± 0.9	9.8 ± 0.9	2.7 ± 0.7

Table S2. Demographics in census tracts covered by GCAS during TRACER-AQ for rasters with >60% tract coverage in the morning (r1: 9–11:30 am LT), midday (r2: 11:30 am–2 pm LT), and afternoon (r3: 2:30–5 pm LT) and across the full MSA.

Date	Raster	Whites	Black and African Americans	Hispanics	Asians
9/1/2021	r1	31%	18%	40%	8%
9/1/2021	r2	29%	18%	43%	7%
9/3/2021	r1	32%	18%	39%	8%
9/3/2021	r2	32%	18%	39%	8%
9/8/2021	r1	31%	18%	40%	8%
9/8/2021	r2	31%	18%	40%	8%
9/8/2021	r3	31%	18%	40%	8%
9/9/2021	r1	31%	18%	40%	8%
9/9/2021	r2	31%	18%	40%	8%
9/9/2021	r3	31%	18%	40%	8%
9/10/2021	r1	31%	18%	40%	8%
9/10/2021	r2	31%	18%	40%	8%
9/10/2021	r3	31%	18%	40%	8%
9/11/2021	r1	31%	18%	40%	8%
9/11/2021	r2	30%	19%	40%	8%
9/23/2021	r1	31%	18%	40%	8%
9/23/2021	r2	32%	18%	40%	8%
9/23/2021	r3	32%	18%	40%	8%
9/24/2021	r1	31%	18%	40%	8%
9/24/2021	r2	32%	18%	40%	8%
9/24/2021	r3	32%	18%	40%	8%
9/25/2021	r1	31%	18%	40%	8%
9/25/2021	r2	31%	18%	40%	8%
9/25/2021	r3	32%	18%	39%	8%
9/26/2021	r1	31%	18%	40%	8%
9/26/2021	r2	31%	18%	40%	8%
9/26/2021	r3	33%	17%	41%	7%
Mean		31%	18%	40%	8%
MSA Demographics		35%	17%	37%	8%

Table S3. Differences in race and ethnicity along census tracts covered during TRACER-AQ on rasters with >60% coverage in the morning (r1: 9–11:30 am LT), midday (r2: 11:30 am–2 pm LT), and afternoon (r3: 2:30–5 pm LT) compared to race and ethnicity across the Houston MSA and UA.

		Houston MSA				Houston UA			
Date	Raster	White	Black and African American	Hispanic and Latino	Asian	White	Black and African American	Hispanic and Latino	Asian
9/1/2021	r1	–11%	10%	6%	2%	4%	1%	–1%	–10%
9/1/2021	r2	–18%	8%	15%	–7%	–3%	0%	7%	–18%
9/3/2021	r1	–8%	8%	4%	0%	8%	–1%	–3%	–12%
9/3/2021	r2	–10%	7%	6%	2%	6%	–1%	–2%	–10%
9/8/2021	r1	–12%	10%	6%	2%	4%	1%	–1%	–10%
9/8/2021	r2	–12%	10%	6%	2%	4%	1%	–1%	–10%
9/8/2021	r3	–11%	10%	6%	1%	4%	1%	–1%	–10%
9/9/2021	r1	–11%	9%	6%	1%	4%	1%	–1%	–10%
9/9/2021	r2	–12%	10%	6%	2%	4%	1%	–1%	–10%
9/9/2021	r3	–11%	10%	6%	1%	4%	1%	–1%	–11%
9/10/2021	r1	–12%	10%	6%	2%	4%	1%	–1%	–10%
9/10/2021	r2	–12%	10%	6%	2%	4%	1%	–1%	–10%
9/10/2021	r3	–11%	10%	6%	2%	4%	1%	–1%	–10%
9/11/2021	r1	–11%	10%	6%	2%	4%	1%	–1%	–10%
9/11/2021	r2	–15%	13%	7%	6%	0%	4%	0%	–6%
9/23/2021	r1	–11%	9%	6%	2%	4%	1%	–1%	–10%
9/23/2021	r2	–11%	9%	6%	2%	5%	0%	–1%	–10%
9/23/2021	r3	–11%	9%	6%	2%	4%	0%	–1%	–10%
9/24/2021	r1	–12%	9%	6%	2%	4%	1%	–1%	–10%
9/24/2021	r2	–11%	9%	6%	2%	5%	1%	–1%	–10%
9/24/2021	r3	–11%	9%	6%	2%	5%	1%	–1%	–10%
9/25/2021	r1	–12%	9%	6%	2%	4%	1%	–1%	–10%
9/25/2021	r2	–11%	10%	6%	2%	4%	1%	–1%	–10%
9/25/2021	r3	–9%	8%	5%	0%	7%	–1%	–2%	–12%
9/26/2021	r1	–11%	9%	6%	2%	4%	1%	–1%	–10%
9/26/2021	r2	–12%	10%	6%	2%	4%	1%	–1%	–10%
9/26/2021	r3	–8%	4%	9%	–14%	9%	–5%	1%	–25%
Mean		–11%	9%	6%	1%	4%	0%	–1%	–11%

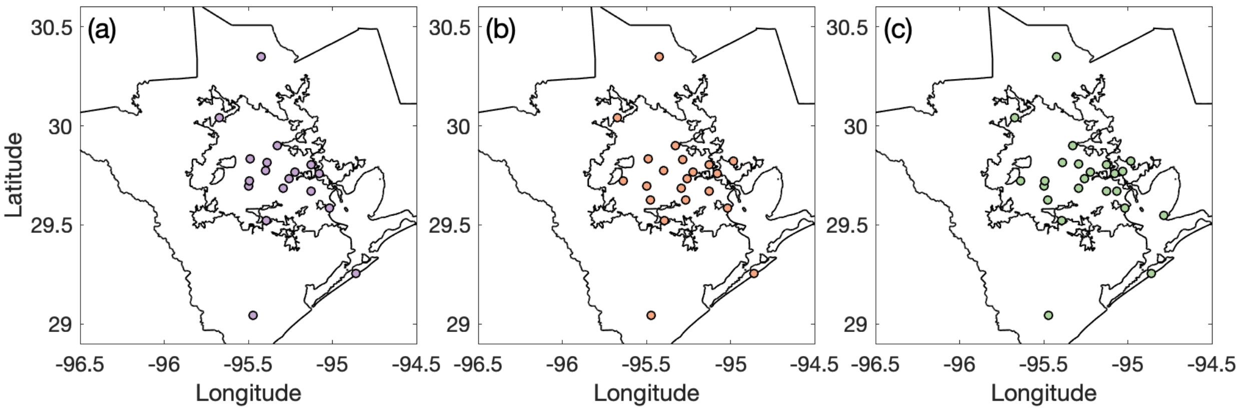

Figure S2. Routine surface monitor locations for NO_2* (a), O_3 (b), and surface meteorology (c).

Equation S1. Population-weighted NO_2 columns are calculated as the product of the census tract averaged NO_2 TVCD ($NO_{2,j}$) and demographic group population (p_j) in the ith tract summed over all tracts with NO_2 data (n). The summation is divided by the demographic group population (p_j).

(Eq. S1) Population-weighted $NO_{2,j} = \sum^{n} NO_{2,i}\, p_{i,j} / \sum^{n} p_{i,j}$

Table S4. Mean daily inequalities (May 2018–November 2022) by TROPOMI pixel size. The pixel size thresholds are defined according to the pixel size quintiles. Uncertainties are 95% confidence intervals from bootstrapped distributions sampled with replacement 10^4 times.

UA						
	Pixel Size Thresholds					Identified Pixel Area Threshold
	All Days	<53 km^2	<41 km^2	<29 km^2	<24 km^2	
Black and African Americans	9 ± 1	9 ± 1	9 ± 1	9 ± 1	9 ± 1	None
Hispanics and Latinos	16 ± 1	17 ± 1	18 ± 1	17 ± 1	17 ± 1	None
Asians	–1 ± 1	–1 ± 1	–1 ± 1	–2 ± 1	–2 ± 2	None
MSA						
	Pixel Size Thresholds					Identified Pixel Area Threshold
	All Days	<54 km^2	<42 km^2	<30 km^2	<24 km^2	
Black and African Americans	17 ± 1	17 ± 1	18 ± 1	17 ± 1	18 ± 2	None
Hispanics and Latinos	24 ± 1	24 ± 1	25 ± 1	24 ± 1	24 ± 2	None
Asians	10 ± 1	9 ± 1	9 ± 1	8 ± 2	9 ± 2	None

Table S5. Mean daily inequalities (May 2018–November 2022) by TROPOMI coverage, defined as the percentage of census tracts with observations. Uncertainties are 95% confidence intervals from bootstrapped distributions sampled with replacement 10^4 times.

UA						
		Inequalities by Census Tract Coverage Level				Selected or Identified Coverage Threshold
	all days	>20% coverage	>40% coverage	>60% coverage	>80% coverage	
Black and African Americans	8 ± 1	9 ± 1	9 ± 1	9 ± 1	8 ± 1	>20%
Hispanics and Latinos	15 ± 1	16 ± 1	17 ± 1	17 ± 1	17 ± 1	>20%
Asians	0 ± 1	–1 ± 1	–1 ± 1	–1 ± 1	–2 ± 1	>20%
**MSA**						
		Inequalities by Census Tract Coverage Level				Selected or Identified Coverage Threshold
	all days	>20% coverage	>40% coverage	>60% coverage	>80% coverage	
Black and African Americans	15 ± 1	17 ± 1	17 ± 1	18 ± 1	18 ± 1	>20%
Hispanics and Latinos	20 ± 1	23 ± 1	24 ± 1	24 ± 1	25 ± 1	>40%
Asians	9 ± 1	10 ± 1	9 ± 1	9 ± 1	9 ± 1	>20%

Table S6. Oversampled versus mean daily NO_2 inequalities on days classified in each orbit pattern. Orbit patterns are identified as overpasses occurring every 16 days from August 2019 to November 2022. Mean inequalities are calculated from the mean of individual census tract-averaged maps. Uncertainties are expressed as standard mean errors. Note: orbit number 11 did not cover the full Houston UA and was not included in the table in the main text.

Relative Inequality (%)						
	Oversampled (0.01° x 0.01°)			Mean Daily Map		
Orbit Pattern	Black and African Americans	Hispanics and Latinos	Asians	Black and African Americans	Hispanics and Latinos	Asians
1	10 ± 1	19 ± 1	2 ± 1	7 ± 1	18 ± 1	–3 ± 1
2	9 ± 1	16 ± 1	1 ± 1	9 ± 1	17 ± 1	–2 ± 1
3	7 ± 1	17 ± 1	0 ± 1	7 ± 1	18 ± 1	–1 ± 2
4	9 ± 1	16 ± 1	0 ± 1	13 ± 1	22 ± 1	1 ± 2
5	7 ± 1	13 ± 1	2 ± 1	8 ± 1	14 ± 1	4 ± 1
6	8 ± 1	16 ± 1	–2 ± 1	8 ± 1	17 ± 1	–1 ± 1
7	8 ± 1	17 ± 1	–1 ± 1	8 ± 1	17 ± 1	–4 ± 1
8	10 ± 1	17 ± 1	3 ± 1	10 ± 1	17 ± 1	1 ± 1
9	9 ± 1	18 ± 1	0 ± 1	11 ± 1	18 ± 1	4 ± 1
10	9 ± 1	15 ± 1	4 ± 1	11 ± 1	16 ± 1	6 ± 1
11	10 ± 1	10 ± 1	6 ± 1	11 ± 1	9 ± 1	9 ± 1
12	11 ± 1	20 ± 1	1 ± 1	9 ± 1	20 ± 1	–0 ± 1
13	11 ± 1	19 ± 1	1 ± 1	10 ± 1	20 ± 1	–4 ± 1
14	8 ± 1	17 ± 1	–3 ± 1	8 ± 1	19 ± 1	–5 ± 1
15	9 ± 1	16 ± 1	1 ± 1	12 ± 1	21 ± 1	2 ± 1
16	10 ± 1	11 ± 1	8 ± 1	11 ± 1	17 ± 1	7 ± 1
Absolute Inequality (x 10^{14} molecules cm^{-2})						
	Oversampled (0.01° x 0.01°)			Mean Daily Map		
Orbit Pattern	Black and African Americans	Hispanics and Latinos	Asians	Black and African Americans	Hispanics and Latinos	Asians
1	3.2 ± 0.4	6.2 ± 0.4	0.5 ± 0.4	2.7 ± 0.5	7.5 ± 0.5	–1.0 ± 0.5
2	3.0 ± 0.4	5.5 ± 0.4	0.3 ± 0.4	3.6 ± 0.4	6.6 ± 0.4	–0.6 ± 0.4
3	2.2 ± 0.4	5.3 ± 0.4	0.1 ± 0.4	2.6 ± 0.5	6.8 ± 0.5	–0.3 ± 0.5
4	2.8 ± 0.3	5.1 ± 0.3	0.1 ± 0.4	5.1 ± 0.6	9.3 ± 0.6	0.3 ± 0.6
5	2.1 ± 0.3	4.3 ± 0.3	0.6 ± 0.3	3.1 ± 0.4	5.6 ± 0.5	1.7 ± 0.5
6	2.2 ± 0.3	4.8 ± 0.3	–0.5 ± 0.3	2.8 ± 0.4	5.7 ± 0.4	–0.4 ± 0.4
7	2.3 ± 0.3	4.9 ± 0.3	–0.3 ± 0.3	2.4 ± 0.3	5.8 ± 0.3	–1.3 ± 0.3
8	3.0 ± 0.3	5.2 ± 0.3	1.0 ± 0.3	3.8 ± 0.4	6.9 ± 0.4	0.5 ± 0.5
9	3.0 ± 0.4	5.9 ± 0.4	0.1 ± 0.4	4.6 ± 0.5	7.6 ± 0.5	1.4 ± 0.5
10	3.0 ± 0.3	4.9 ± 0.3	1.2 ± 0.3	4.4 ± 0.4	6.4 ± 0.4	2.2 ± 0.4
11	3.2 ± 0.3	3.3 ± 0.3	1.9 ± 0.3	4.8 ± 0.5	3.8 ± 0.5	3.9 ± 0.5
12	3.3 ± 0.4	6.4 ± 0.4	0.2 ± 0.4	3.4 ± 0.5	7.7 ± 0.5	–0.1 ± 0.5
13	3.5 ± 0.4	6.3 ± 0.4	0.2 ± 0.4	4.2 ± 0.6	8.5 ± 0.6	–1.5 ± 0.5
14	2.6 ± 0.4	6.0 ± 0.4	–0.9 ± 0.4	3.2 ± 0.5	8.0 ± 0.5	–1.9 ± 0.5
15	2.9 ± 0.4	5.7 ± 0.4	0.2 ± 0.4	5.2 ± 0.6	9.6 ± 0.6	0.9 ± 0.6
16	3.1 ± 0.3	3.7 ± 0.3	2.6 ± 0.3	4.7 ± 0.6	7.4 ± 0.5	3.1 ± 0.6

Table S7. Mean daily population-weighted NO_2 by race-ethnicity in the Houston UA (May 2018–November 2022). Uncertainties are 95% confidence intervals from bootstrapped distributions sampled with replacement 10^4 times.

	White	Black and African Americans	Hispanics and Latinos	Asians
Population-weighted NO_2 (x 10^{15} molecules cm^{-2})	3.5 ± 0.1	3.9 ± 0.1	4.2 ± 0.1	3.5 ± 0.1

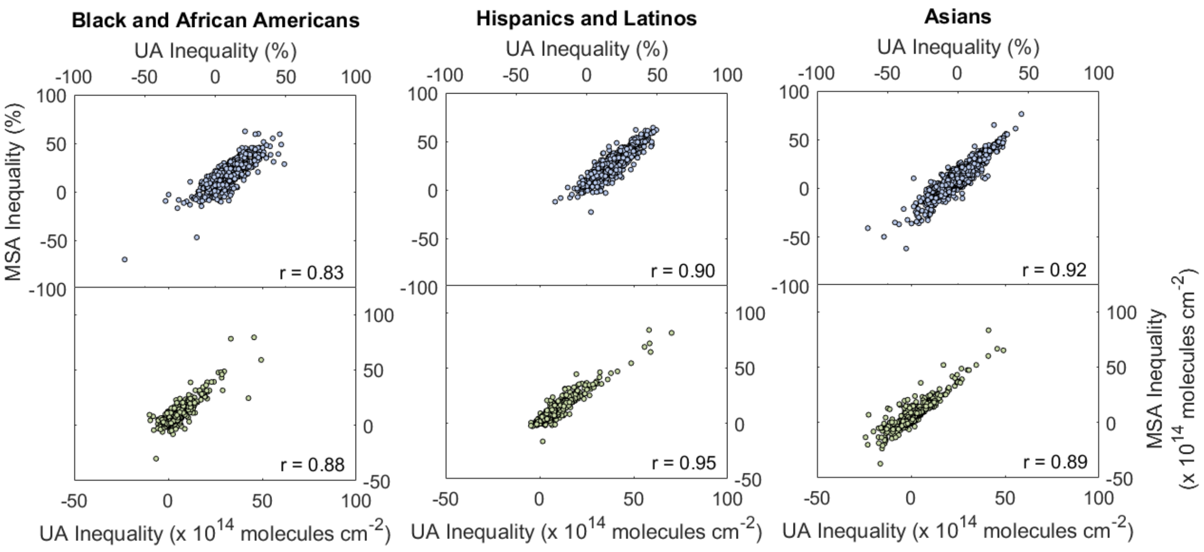

Figure S3. Scatterplots between daily UA and MSA-level relative and absolute inequalities on days meeting metric-specific coverage thresholds for Black and African Americans (a), Hispanics and Latinos (b), and Asians (c). Pearson correlation coefficients are reported in each panel.

SI Appendix 1. Comparing TRACER-AQ and DISCOVER-AQ Inequalities. We compare NO_2 inequalities observed by GCAS during TRACER–AQ (1 September 2021–26 September 2021) and DISCOVER–AQ (4 September 2013–27 September 2013) along spatially coincident census tracts based on 2020 census tract polygons and 2020 ACS demographics. We identify 792 spatially coincident census tracts as tracts covered by the maximum tract coverage flights of both DISCOVER–AQ (4 September 2013 fight 1 raster 1, 864 census tracts) and TRACER–AQ (25 September 2021 raster 3, 1382 tracts).

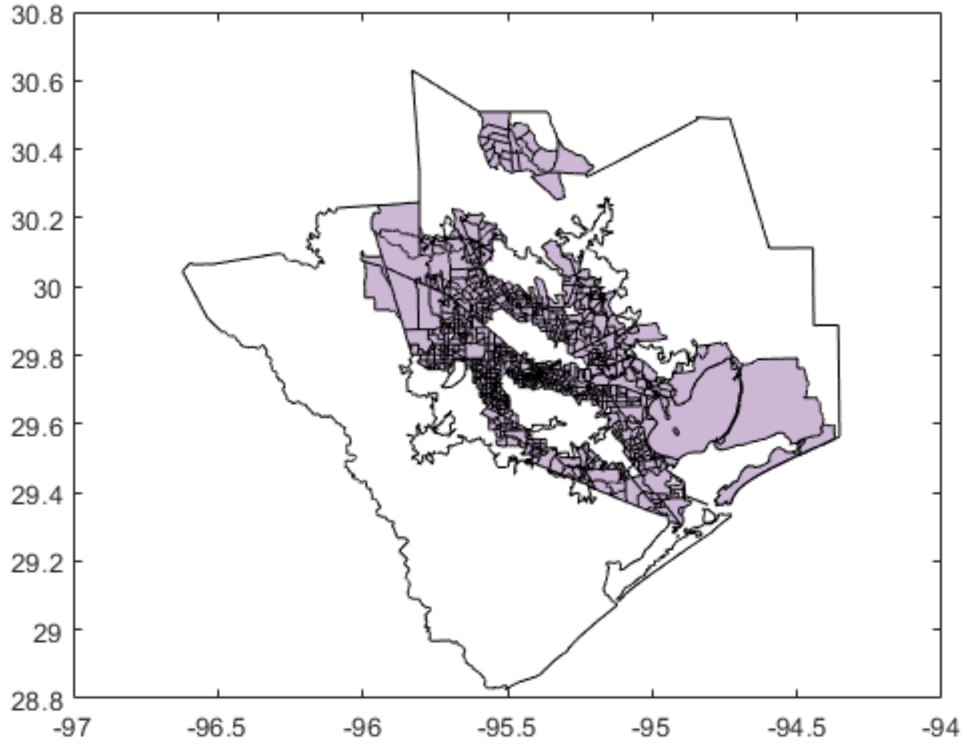

Figure S4. Spatially coincident tracts identified between TRACER-AQ and DISCOVER-AQ flights.

Table S8. Weekday inequalities during TRACER-AQ along spatially coincident census tracts also sampled during DISCOVER-AQ. Spatially coincident census tracts are identified using the two GCAS flights with the greatest tract coverage during DISCOVER-AQ (4 September 2013, morning) and TRACER-AQ (25 September 2021, afternoon). We include inequalities from flights with >60% coverage of coincident census tracts, removing inequalities on 30 August 2021. Mean inequality uncertainties are 95% confidence intervals from bootstrapped distributions sampled with replacement 10^4 times.

Date	Raster	Relative Inequality (%)			Absolute Inequality (x 10^{14} molecules cm^{-2})		
		Black and African Americans	Hispanics and Latinos	Asians	Black and African Americans	Hispanics and Latinos	Asians
9/1/2021	r1	44 ± 5	48 ± 4	10 ± 4	19.9 ± 2.2	22.1 ± 1.9	3.7 ± 1.6
9/1/2021	r2	38 ± 4	43 ± 4	6 ± 3	18.5 ± 1.8	21.5 ± 1.8	2.5 ± 1.3
9/3/2021	r1	39 ± 4	43 ± 4	17 ± 3	23.8 ± 2.5	27.1 ± 2.3	8.9 ± 1.7
9/3/2021	r2	24 ± 4	38 ± 3	7 ± 2	9.7 ± 1.5	16.8 ± 1.4	2.7 ± 0.9
9/8/2021	r1	–9 ± 4	16 ± 4	–15 ± 4	–7.2 ± 3.1	14.4 ± 3.4	–11.2 ± 3.2
9/8/2021	r2	–1 ± 4	18 ± 4	–7 ± 4	–0.3 ± 2.1	11.5 ± 2.3	–3.9 ± 2.1
9/8/2021	r3	–11 ± 3	13 ± 3	–3 ± 3	–6.3 ± 1.9	8.5 ± 2.2	–1.6 ± 2.0
9/9/2021	r1	–19 ± 4	14 ± 4	–14 ± 4	–12.0 ± 2.5	10.7 ± 2.8	–9.2 ± 2.5
9/9/2021	r2	–2 ± 4	–2 ± 4	4 ± 4	–0.9 ± 2.3	–1.2 ± 2.1	2.4 ± 2.4
9/9/2021	r3	–1 ± 3	22 ± 3	5 ± 3	–0.3 ± 1.2	10.8 ± 1.4	2.3 ± 1.2
9/10/2021	r1	17 ± 3	30 ± 3	21 ± 3	12.5 ± 2.4	23.8 ± 2.8	15.9 ± 2.4
9/10/2021	r2	19 ± 5	26 ± 4	26 ± 4	11.5 ± 2.8	16.1 ± 2.5	16.2 ± 2.6
9/10/2021	r3	5 ± 2	25 ± 3	8 ± 2	2.3 ± 1.2	13.6 ± 1.6	4.3 ± 1.2
9/23/2021	r1	10 ± 4	26 ± 4	28 ± 4	12.2 ± 4.8	34.0 ± 4.9	36.1 ± 5.2
9/23/2021	r2	3 ± 4	18 ± 4	18 ± 4	2.7 ± 3.1	16.7 ± 3.3	16.2 ± 3.3
9/23/2021	r3	7 ± 4	25 ± 4	15 ± 4	6.0 ± 3.3	23.4 ± 3.3	12.8 ± 3.4
9/24/2021	r1	10 ± 5	18 ± 4	31 ± 4	12.2 ± 6.0	23.3 ± 5.8	44.4 ± 6.0
9/24/2021	r2	–15 ± 4	3 ± 4	6 ± 4	–12.9 ± 3.2	3.1 ± 3.3	5.4 ± 3.5
9/24/2021	r3	3 ± 4	18 ± 4	13 ± 4	2.3 ± 3.0	15.0 ± 3.0	10.4 ± 3.2
Mean		9 ± 8	24 ± 6	9 ± 6	4.9 ± 4.6	16.4 ± 3.7	9.6 ± 6.2

Table S9. Inequalities during DISCOVER-AQ along spatially coincident census tracts on weekdays. We only include data from flights sampling >60% of coincident tracts between TRACER-AQ and DISCOVER-AQ, removing 6 September 2013 (mid-afternoon flight), 12 September 2013 (morning flight), 14 September 2013 (an afternoon and mid-afternoon flight), 25 September 2013 (morning), and 27 September 2013 (morning). Mean inequality uncertainties are 95% confidence intervals from bootstrapped distributions sampled with replacement 10^4 times.

Date	Flight	Raster	Relative Inequality (%)			Absolute Inequality (x 10^{14} molecules cm^{-2})		
			Black and African Americans	Hispanics and Latinos	Asians	Black and African Americans	Hispanics and Latinos	Asians
9/4/2013	F1	r1	5 ± 4	25 ± 4	–18 ± 4	4.3 ± 3.4	23.0 ± 3.4	–12.9 ± 2.7
9/4/2013	F1	r2	–6 ± 5	35 ± 5	–25 ± 5	–5.5 ± 4.5	39.6 ± 5.3	–20.4 ± 4.1
9/4/2013	F2	r1	21 ± 5	26 ± 4	14 ± 5	19.9 ± 4.4	25.7 ± 4.1	12.6 ± 4.7
9/4/2013	F2	r2	19 ± 2	21 ± 2	14 ± 2	16.0 ± 1.9	18.0 ± 2.0	11.8 ± 1.9
9/6/2013	F1	r1	21 ± 4	16 ± 4	28 ± 3	20.1 ± 4.0	15.0 ± 3.6	28.0 ± 3.5
9/6/2013	F1	r2	16 ± 4	20 ± 3	22 ± 4	10.4 ± 2.5	13.4 ± 2.3	15.2 ± 2.4
9/6/2013	F2	r2	8 ± 4	16 ± 4	16 ± 4	4.8 ± 2.5	10.4 ± 2.7	10.5 ± 2.7
9/11/2013	F1	r1	27 ± 4	26 ± 4	32 ± 4	25.8 ± 4.1	25.0 ± 4.1	31.1 ± 4.0
9/11/2013	F1	r2	–4 ± 6	–5 ± 6	17 ± 6	–3.7 ± 5.3	–4.3 ± 4.7	16.2 ± 5.6
9/11/2013	F2	r1	–5 ± 5	13 ± 5	12 ± 6	–3.2 ± 3.3	8.8 ± 3.3	8.3 ± 3.8
9/11/2013	F2	r2	–6 ± 5	18 ± 5	13 ± 6	–3.4 ± 3.4	12.9 ± 3.6	8.9 ± 3.9
9/12/2013	F1	r1	10 ± 4	21 ± 4	24 ± 3	9.3 ± 3.5	20.0 ± 3.4	23.0 ± 3.3
9/12/2013	F2	r1	14 ± 4	37 ± 5	17 ± 4	7.7 ± 2.3	23.1 ± 2.8	9.4 ± 2.3
9/12/2013	F2	r2	13 ± 6	27 ± 5	22 ± 6	9.5 ± 4.1	21.6 ± 4.1	17.0 ± 4.3
9/13/2013	F1	r1	–7 ± 5	17 ± 5	2 ± 5	–7.1 ± 5.3	18.8 ± 5.1	1.9 ± 5.4
9/13/2013	F1	r2	–2 ± 5	15 ± 4	15 ± 5	–1.9 ± 5.0	15.2 ± 4.6	15.5 ± 5.3
9/13/2013	F2	r1	–1 ± 5	31 ± 5	9 ± 5	–0.3 ± 2.6	21.4 ± 3.3	5.2 ± 2.8
9/13/2013	F2	r2	4 ± 4	35 ± 4	12 ± 4	2.4 ± 2.5	25.3 ± 3.1	7.8 ± 2.5
9/18/2013	F1	r1	4 ± 5	10 ± 4	21 ± 4	3.5 ± 3.6	8.5 ± 3.6	18.9 ± 3.6
9/18/2013	F1	r2	–4 ± 5	4 ± 5	17 ± 5	–3.3 ± 3.6	3.4 ± 3.9	15.0 ± 4.4
9/18/2013	F2	r1	2 ± 3	5 ± 3	11 ± 3	1.5 ± 1.8	3.3 ± 1.8	7.1 ± 1.8
9/18/2013	F2	r2	9 ± 3	8 ± 3	9 ± 3	5.7 ± 1.9	5.3 ± 1.9	6.3 ± 1.7
9/24/2013	F1	r1	–12 ± 4	–1 ± 4	8 ± 4	–9.7 ± 3.3	–0.7 ± 3.2	7.0 ± 3.8
9/24/2013	F1	r2	–1 ± 4	–5 ± 4	4 ± 4	–0.9 ± 2.4	–2.7 ± 2.1	2.4 ± 2.3
9/24/2013	F2	r1	–12 ± 3	7 ± 3	–4 ± 4	–5.6 ± 1.6	3.5 ± 1.7	–1.8 ± 1.8
9/24/2013	F2	r2	–14 ± 4	5 ± 3	–5 ± 4	–8.1 ± 2.0	3.1 ± 2.1	–3.1 ± 2.1
9/25/2013	F1	r1	7 ± 6	40 ± 5	–7 ± 6	10.6 ± 8.6	74.1 ± 9.0	–10.6 ± 7.9
9/25/2013	F2	r1	–16 ± 5	18 ± 5	–18 ± 5	–15.2 ± 4.3	19.6 ± 5.0	–16.8 ± 4.7
9/25/2013	F2	r2	–17 ± 4	19 ± 4	–21 ± 4	–18.0 ± 4.6	23.2 ± 5.0	–22.0 ± 4.5
9/26/2013	F1	r1	45 ± 5	53 ± 4	10 ± 4	64.8 ± 7.3	80.0 ± 6.5	11.5 ± 4.3
9/26/2013	F1	r2	34 ± 5	30 ± 4	6 ± 4	47.0 ± 6.2	40.5 ± 4.8	7.2 ± 4.8
9/26/2013	F2	r1	39 ± 4	45 ± 4	10 ± 3	28.7 ± 3.1	34.5 ± 2.8	6.0 ± 1.9
9/26/2013	F2	r2	37 ± 4	40 ± 4	8 ± 4	31.0 ± 3.6	35.1 ± 3.2	5.8 ± 2.6
9/27/2013	F1	r1	34 ± 6	14 ± 6	25 ± 6	39.9 ± 7.2	14.3 ± 6.0	27.4 ± 6.7
Mean			8 ± 6	20 ± 5	8 ± 5	9.1 ± 6.1	21.2 ± 6.4	7.1 ± 4.3
Mean without 9/25			10 ± 6	20 ± 5	11 ± 4	10.9 ± 6.3	19.3 ± 5.9	9.4 ± 3.7

Table S10. Demographics and number of census tracts covered along spatially coincident weekday flights during DISCOVER-AQ compared to TRACER-AQ. The differences in demographics with respect to the demographics across the urban area and metro area are reported.

			Demographics Covered				Difference in Demographics Compared to the UA				Difference in Demographics Compared to the MSA			
Date	Raster	Flight	Whites	Black and African Americans	Hispanics	Asians	Whites	Black and African Americans	Hispanics	Asians	Whites	Black and African Americans	Hispanics	Asians
9/4/2013	r1	F1	33%	17%	40%	7%	10%	–6%	0%	–25%	–6%	3%	8%	–15%
9/4/2013	r2	F1	32%	17%	42%	6%	8%	–7%	3%	–27%	–8%	1%	11%	–17%
9/4/2013	r1	F2	33%	17%	41%	6%	9%	–5%	2%	–29%	–8%	4%	10%	–19%
9/4/2013	r2	F2	33%	18%	41%	6%	8%	–4%	2%	–27%	–8%	4%	9%	–18%
9/6/2013	r1	F1	33%	17%	41%	7%	9%	–5%	1%	–24%	–7%	3%	8%	–14%
9/6/2013	r2	F1	33%	17%	41%	6%	8%	–5%	3%	–27%	–8%	3%	10%	–17%
9/6/2013	r2	F2	35%	17%	40%	7%	15%	–9%	–2%	–25%	–2%	–1%	6%	–15%
9/11/2013	r1	F1	33%	17%	41%	7%	9%	–7%	1%	–22%	–7%	1%	8%	–12%
9/11/2013	r2	F1	34%	17%	40%	7%	12%	–9%	1%	–23%	–5%	–1%	8%	–13%
9/11/2013	r1	F2	34%	17%	40%	7%	11%	–7%	0%	–24%	–5%	1%	7%	–13%
9/11/2013	r2	F2	32%	17%	41%	7%	8%	–6%	3%	–25%	–8%	2%	10%	–15%
9/12/2013	r1	F1	33%	17%	41%	7%	8%	–7%	2%	–24%	–8%	1%	10%	–14%
9/12/2013	r1	F2	33%	17%	41%	7%	8%	–7%	3%	–25%	–8%	1%	10%	–15%
9/12/2013	r2	F2	33%	17%	41%	7%	8%	–7%	2%	–24%	–8%	1%	10%	–13%
9/13/2013	r1	F1	35%	15%	41%	6%	16%	–16%	2%	–28%	–2%	–9%	9%	–18%
9/13/2013	r2	F1	35%	16%	41%	6%	15%	–15%	2%	–28%	–2%	–8%	9%	–18%
9/13/2013	r1	F2	34%	15%	41%	6%	14%	–16%	3%	–29%	–3%	–8%	10%	–19%
9/13/2013	r2	F2	35%	15%	41%	6%	16%	–16%	1%	–27%	–1%	–8%	8%	–18%
9/18/2013	r1	F1	35%	15%	41%	6%	15%	–16%	3%	–29%	–2%	–9%	10%	–20%
9/18/2013	r2	F1	35%	15%	42%	6%	15%	–20%	4%	–28%	–3%	–13%	12%	–18%
9/18/2013	r1	F2	34%	16%	42%	6%	12%	–15%	4%	–28%	–5%	–8%	12%	–18%
9/18/2013	r2	F2	32%	16%	43%	6%	7%	–11%	6%	–27%	–9%	–3%	14%	–18%
9/24/2013	r1	F1	35%	16%	41%	6%	15%	–15%	2%	–27%	–3%	–8%	9%	–18%
9/24/2013	r2	F1	35%	16%	41%	6%	15%	–13%	1%	–28%	–3%	–5%	8%	–18%
9/24/2013	r1	F2	35%	15%	41%	6%	15%	–16%	1%	–27%	–2%	–8%	9%	–17%
9/24/2013	r2	F2	35%	16%	40%	7%	16%	–15%	0%	–26%	–2%	–7%	8%	–16%
9/25/2013	r1	F1	34%	16%	41%	6%	14%	–15%	2%	–27%	–3%	–8%	10%	–18%
9/25/2013	r1	F2	35%	16%	41%	6%	15%	–15%	2%	–29%	–3%	–8%	10%	–19%
9/25/2013	r2	F2	33%	16%	42%	6%	9%	–12%	5%	–28%	–8%	–4%	13%	–19%
9/26/2013	r1	F1	35%	16%	41%	6%	15%	–14%	1%	–28%	–2%	–6%	8%	–18%
9/26/2013	r2	F1	35%	16%	41%	6%	15%	–15%	3%	–32%	–2%	–8%	10%	–23%
9/26/2013	r1	F2	35%	16%	40%	6%	16%	–14%	1%	–29%	–1%	–6%	8%	–19%
9/26/2013	r2	F2	34%	16%	41%	6%	13%	–15%	3%	–27%	–4%	–7%	11%	–17%
9/27/2013	r1	F1	34%	16%	42%	6%	12%	–14%	5%	–33%	–5%	–6%	13%	–24%
Mean			34%	16%	41%	6%	12%	–12%	2%	–27%	–5%	–4%	10%	–17%

Table S11. Demographics and number of census tracts covered along spatially coincident weekday flights during TRACER-AQ compared to DISCOVER-AQ. The differences in demographics with respect to the demographics across the urban area and metro area are reported.

		Demographics				Demographics Compared to Urban Area				Demographics Compared to MSA			
Date	Raster	Whites	Black and African Americans	Hispanics and Latinos	Asians	Whites	Black and African Americans	Hispanics and Latinos	Asians	Whites	Black and African Americans	Hispanics and Latinos	Asians
9/1/2021	r1	33%	18%	41%	7%	8%	–4%	1%	–24%	–8%	4%	8%	–14%
9/1/2021	r2	32%	16%	43%	7%	5%	–10%	6%	–22%	–11%	–2%	14%	–11%
9/3/2021	r1	33%	17%	40%	7%	11%	–6%	0%	–25%	–6%	2%	8%	–15%
9/3/2021	r2	32%	17%	42%	6%	6%	–5%	4%	–27%	–10%	3%	12%	–18%
9/8/2021	r1	33%	17%	41%	7%	8%	–4%	1%	–24%	–8%	4%	9%	–14%
9/8/2021	r2	33%	18%	40%	7%	8%	–4%	1%	–24%	–8%	4%	8%	–14%
9/8/2021	r3	33%	17%	41%	7%	8%	–4%	1%	–24%	–8%	4%	9%	–14%
9/9/2021	r1	33%	17%	41%	7%	8%	–4%	1%	–24%	–8%	4%	9%	–14%
9/9/2021	r2	33%	18%	40%	7%	8%	–4%	1%	–24%	–8%	4%	8%	–14%
9/9/2021	r3	33%	17%	41%	7%	8%	–4%	1%	–24%	–8%	4%	9%	–14%
9/10/2021	r1	33%	18%	40%	7%	8%	–4%	1%	–24%	–8%	4%	8%	–14%
9/10/2021	r2	33%	18%	40%	7%	8%	–4%	1%	–24%	–8%	4%	8%	–14%
9/10/2021	r3	33%	18%	40%	7%	8%	–4%	1%	–24%	–8%	4%	8%	–14%
9/23/2021	r1	33%	17%	41%	7%	8%	–4%	1%	–24%	–8%	4%	9%	–14%
9/23/2021	r2	33%	18%	40%	7%	8%	–4%	1%	–24%	–8%	4%	8%	–14%
9/23/2021	r3	33%	18%	40%	7%	8%	–4%	1%	–24%	–8%	4%	8%	–14%
9/24/2021	r1	33%	17%	41%	7%	8%	–4%	1%	–24%	–8%	4%	9%	–14%
9/24/2021	r2	33%	18%	40%	7%	8%	–4%	1%	–24%	–8%	4%	8%	–14%
9/24/2021	r3	33%	18%	40%	7%	8%	–4%	1%	–24%	–8%	4%	8%	–14%
Mean		33%	17%	41%	7%	8%	–5%	1%	–24%	–8%	4%	9%	–14%

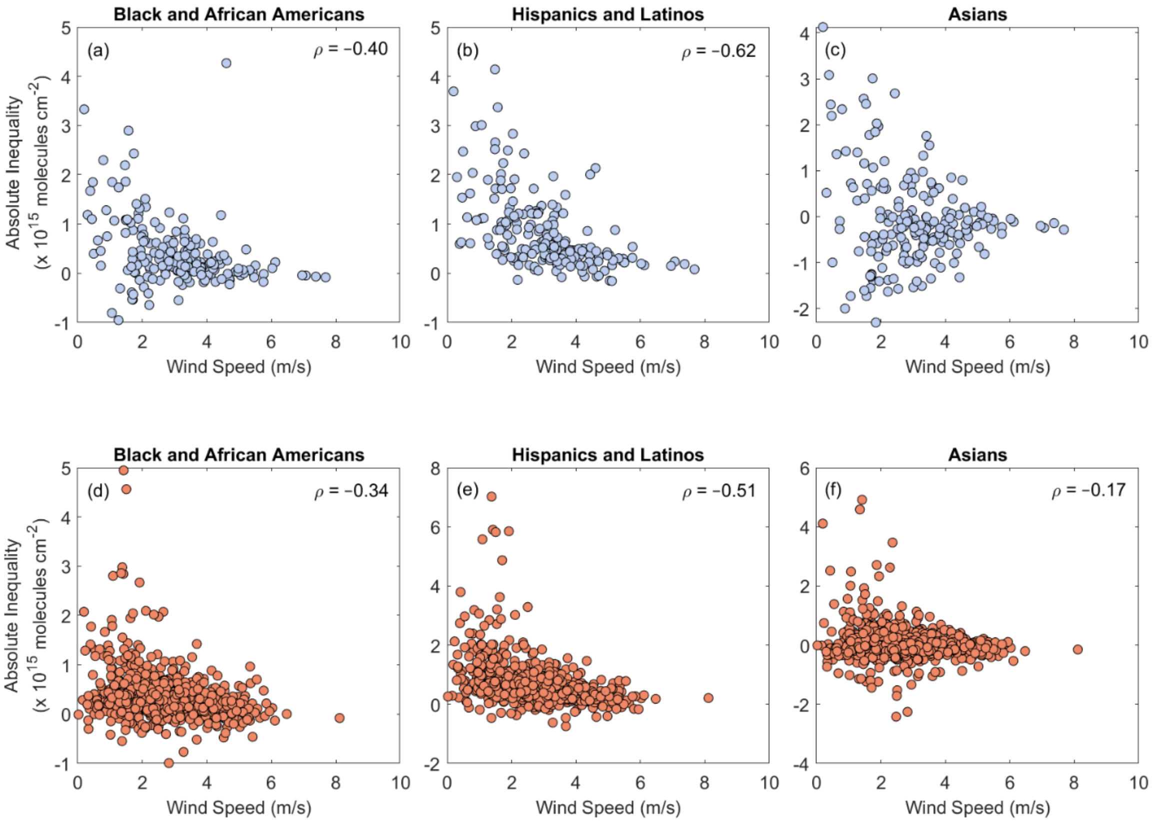

Figure S5. Scatter plots of absolute NO_2 inequalities and surface wind speed during winter months (December–February) (a–c) and during ozone season (March–November) (d–f) for Black and African Americans, Hispanics and Latinos, and Asians. Spearman rank correlation coefficients are reported in each panel when $p < 0.05$.

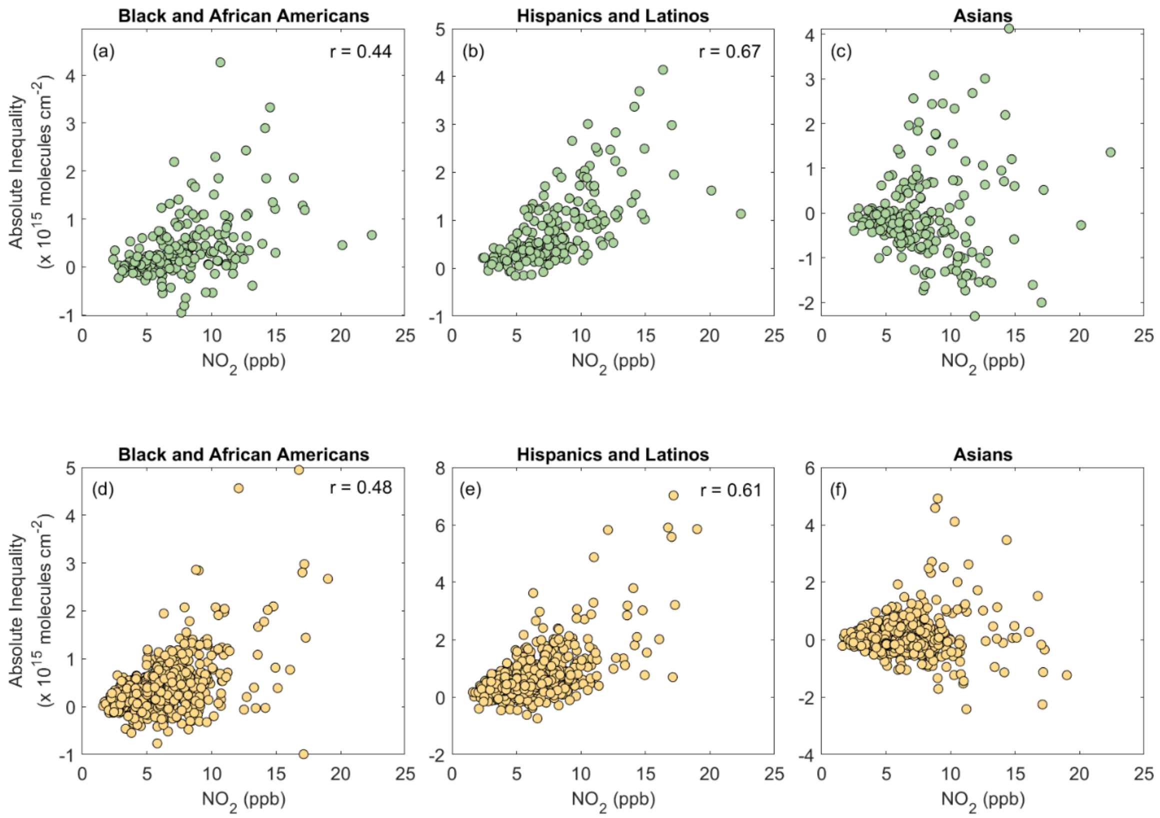

Figure S6. Scatter plots of absolute NO_2 inequalities and surface NO_2* during winter months (December–February) (a–c) and during ozone season (March–November) (d–f) for Black and African Americans, Hispanics and Latinos, and Asians. Pearson correlation coefficients are reported in each panel when $p < 0.05$.

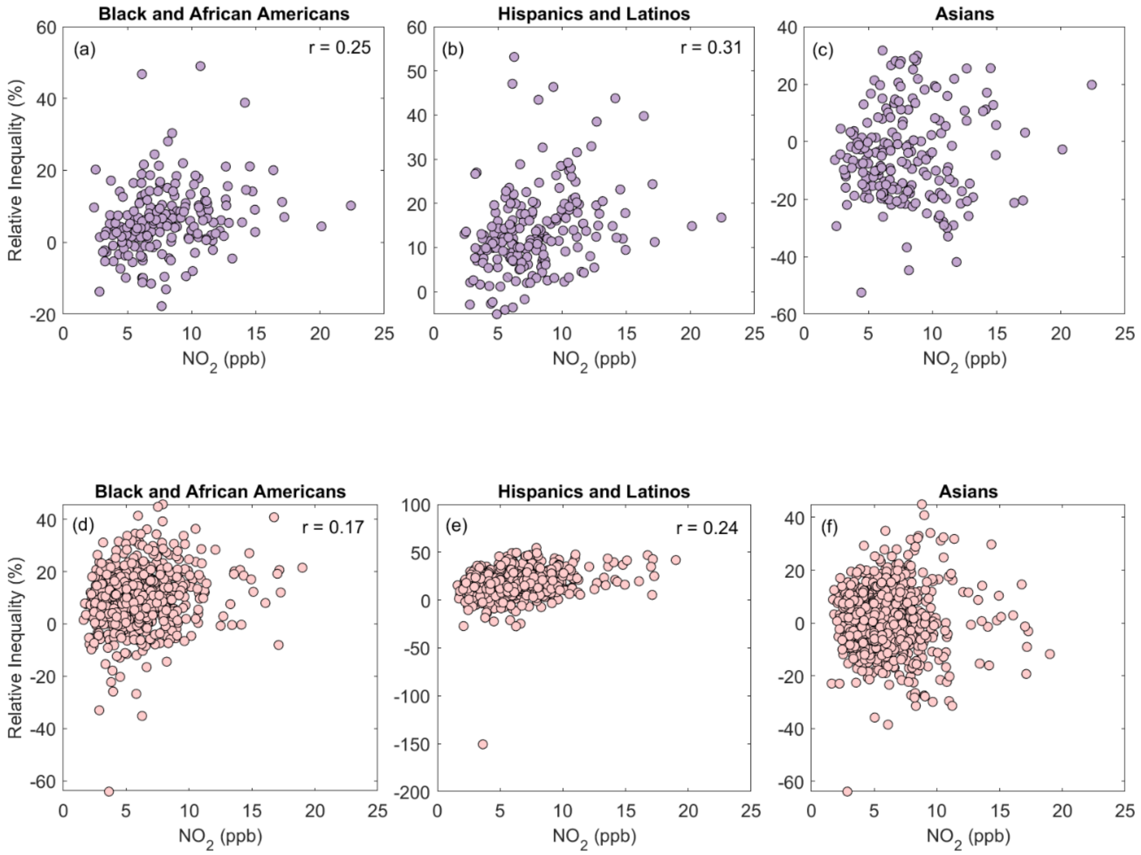

Figure S7. Scatter plots of relative NO_2 inequalities and surface NO_2* during winter months (December–February) (a–c) and during ozone season (March–November) (d–f) for Black and African Americans, Hispanics and Latinos, and Asians. Pearson correlation coefficients are reported in each panel when $p < 0.05$.

SI Appendix 2: Technical Details: Generalized Additive Model (GAM). Regression models enable predictive analysis, reveal classification rules, and provide data-analytic tools for understanding the interactions between different variables. Linear regression models of Y_i and X_i using one explanatory variable are expressed by: $Y_i = \alpha + \beta \times X_i + \varepsilon_i$, where $\varepsilon_i \sim N(0, \sigma^2)$ and α and β are the unknown intercept and slope. Linear models fail when relationships are nonlinear and nonmonotonic, e.g., the PO_3 dependence on NO_2; therefore, we use a general additive model (GAM), which provides better performance for nonlinear relationships and is more appropriate for limited sample sizes than neural networks. In additive modeling, the relationship between Y_i and X_i is expressed by: $Y_i = \alpha + f(X_i) + \varepsilon_i$, where $\varepsilon_i \sim N(0, \sigma^2)$. Here: Y_i is the response variable; X_i is the explanatory variable; $f(X_i)$ is the smoothing curve; ε_i is the residual to capture unexplained information, with the assumption data are normally distributed with expectation 0 and variance σ^2; and α is the unknown intercept.[2-4]

We use R to construct the GAM, testing the 'gam' and 'mgcv' packages on NO_2 inequalities calculated using TROPOMI NO_2 reprocessed on the S5P-PAL system from May 2018–September 2021 and temperature ranges based on quintiles. These data are different than in the final version of the paper but should not substantially affect our decisions based on this evaluation. In the 'gam' package, a back-fitting algorithm was used to estimate one smoother at a time. The 'mgcv' package allowed for cross-validation and generalized mixing modeling. We test a LOESS and spline smoother in 'gam' package and cubic spline smoother in 'mgcv' package. The core R code of each method is shown in Table S12. By using LOESS in the 'gam' package, we fit a local polynomial model of order two and set the percentage of the data inside the window as 0.9 to avoid over-wiggling (Figure S8). We fit a smoothing spline using the 'gam' package with three degrees of freedom (df) (Figure S9), which provides the best performance out of 2–10 df based on the Akaike

Information Criteria (AIC), a measure of goodness of fit and model complexity (Table S13). Details on the parameters in the core code are provided in Hastie.[5] We used the cubic regression spline function in the 'mgcv' package to fit gam using cross-validation to estimate the optimal amount of smoothing (Figure S10). Briefly, 'mgcv' divided X into different intervals and fit a cubic polynomial ($Y_i = \alpha + \beta_1 \times X_i + \beta_2 \times X_i^2 + \beta_3 \times X_i^3$) in each interval that was used to construct the smoothing curve.

We check the models for homogeneity and normality, as shown in Figures S11–S13 and S14–16, respectively. Heterogeneity occurs when the data spread is not equal at each X value and can be identified by plotting the residuals against fitted values. We opt against transforming the data, as there is no extreme heterogeneity. We generate QQ-plots of the residuals, finding all metrics have strong normality.[3] Finally, we compare the AIC value of all these methods (Table S13). While the AIC are similar, the cubic spline smoother in 'mgcv' package generally gives the best performance, followed by the spline smoother in 'gam' package. However, 'mgcv' over-wiggles in some cases, and because of this, we use the spline smoother in the 'gam' package with 3 df as our model.

Table S12. Three initial methods tested for GAM construction.

Package	Smoother	Code
gam	loess	gam(O_3 ~ lo(NO_2 absolute inequality, span = 0.9), data = Houston)
gam	spline	gam(O_3 ~ s(NO_2 absolute inequality, 3), data = Houston)
mgcv	cubic spline	gam(O_3 ~ s(NO_2 absolute inequality, fx = FALSE, k=—1, bs = "cr"), data = Houston)

Table S13. AIC values of the three methods based on absolute inequalities for each race-ethnicity group.

	High Temperature	Moderate Temperature	Low Temperature
‘gam’ Package		LOESS	
Black and African Americans	–1174.75	–1184.30	–962.41
Hispanics and Latinos	–1225.91	–1207.14	–970.99
Asians	–1174.13	–1163.95	–938.37
‘gam’ Package		Spline Smoother	
Black and African Americans	–1174.77	–1185.00	–962.46
Hispanics and Latinos	–1226.12	–1208.85	–971.10
Asians	–1173.37	–1163.71	–938.56
‘mgcv’ Package		Cubic Spline Smoother	
Black and African Americans	–1177.02	–1186.11	–963.18
Hispanics and Latinos	–1226.66	–1212.51	–971.58
Asians	–1174.79	–1165.65	–941.24

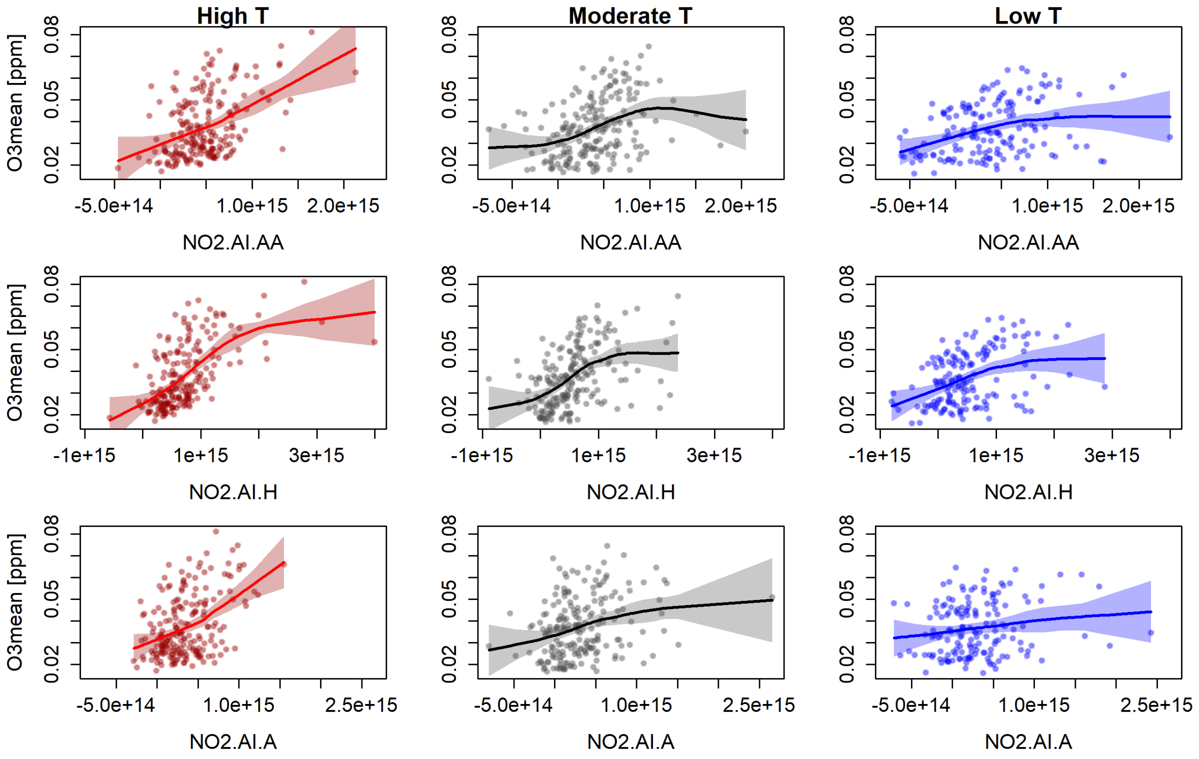

Figure S8. MSA-level mean MDA8 O_3 with fitted value for NO_2 inequalities for Black and African Americans (top row), Hispanics and Latinos (second row), and Asians (third row) using LOESS in the 'gam' package under high (red), moderate (black), and low temperatures (blue). The envelopes are the 95% confidence intervals.

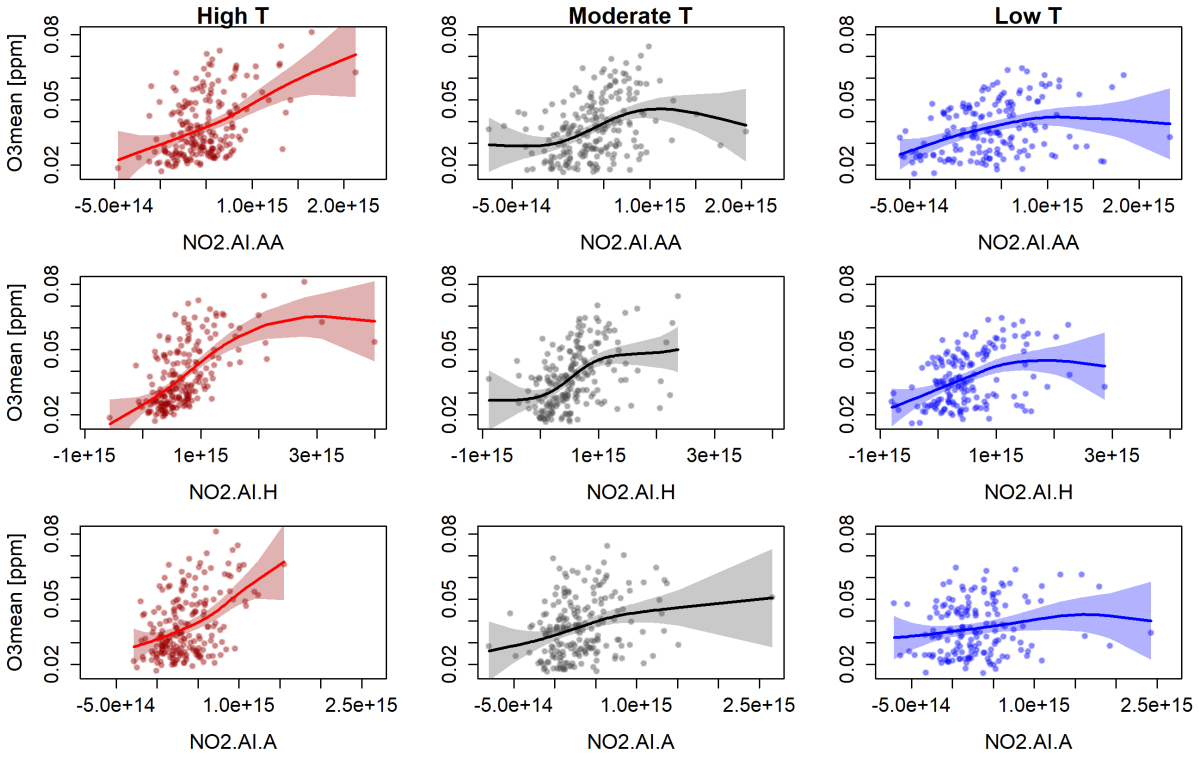

Figure S9. MSA-level mean MDA8 O_3 with fitted value for NO_2 inequalities for Black and African Americans (top row), Hispanics and Latinos (second row), and Asians (third row) using spline smoother in 'gam' package under high (red), moderate (black), and low temperatures (blue). The envelopes are the 95% confidence intervals.

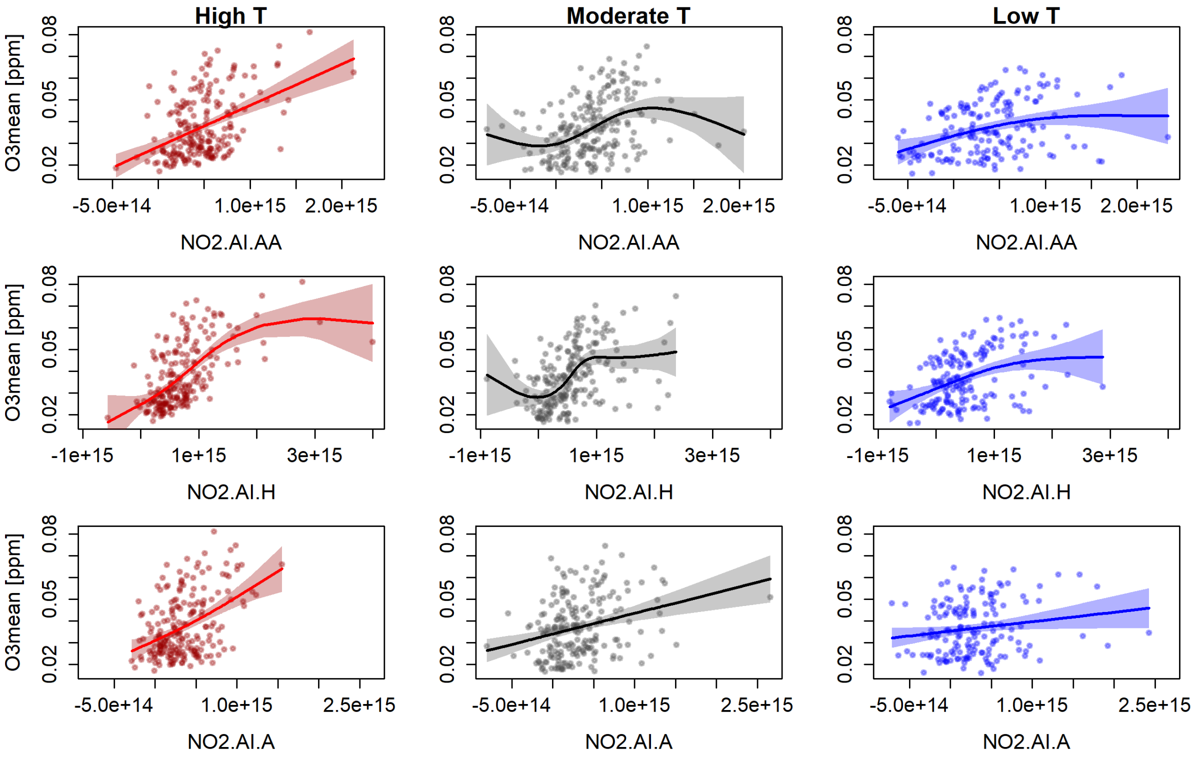

Figure S10. MSA-level mean MDA8 O_3 with fitted value for NO_2 inequalities for Black and African Americans (top row), Hispanics and Latinos (second row), and Asians (third row) using cubic spline smoother in 'mgcv' package under high (red), moderate (black), and low temperatures (blue). The envelopes are the 95% confidence intervals.

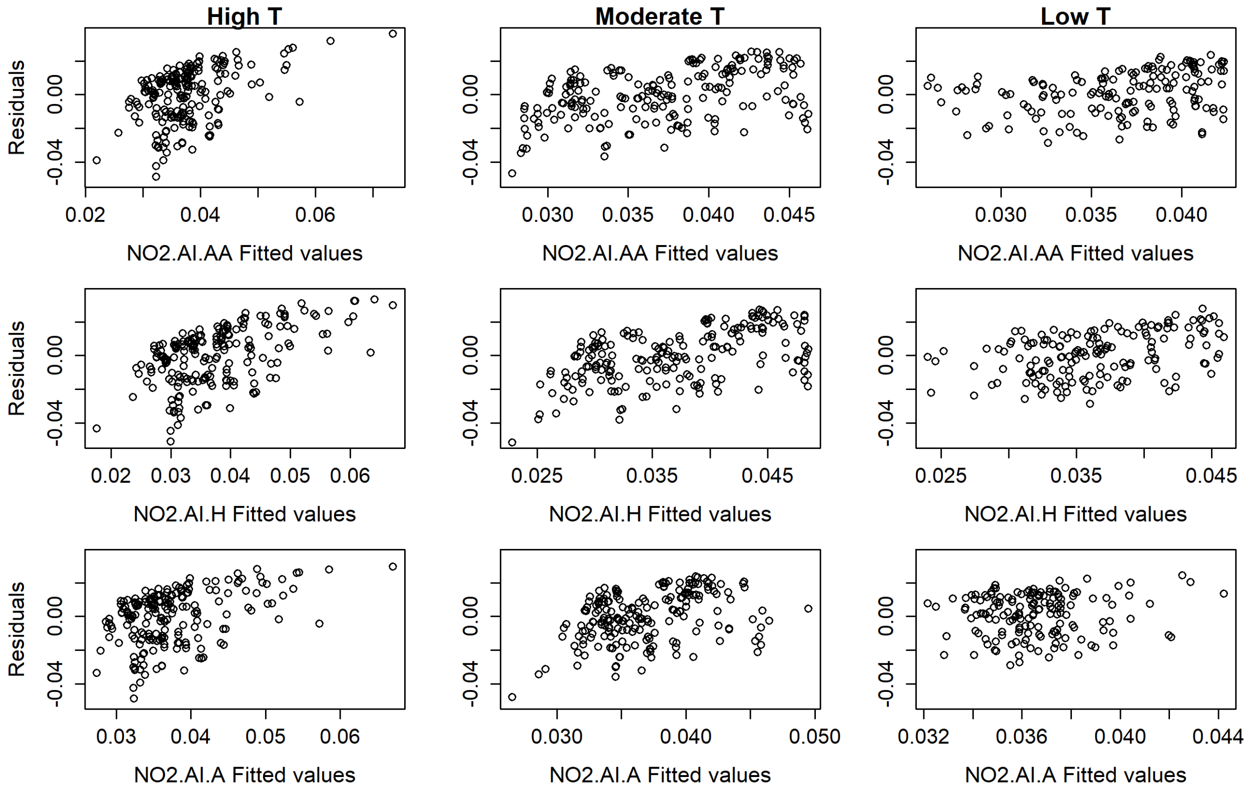

Figure S11. Homogeneity of GAMs for MSA-level mean MDA8 O_3 and NO_2 inequalities for Black and African Americans (top row), Hispanics and Latinos (second row), and Asians (third row) using LOESS in 'gam' package.

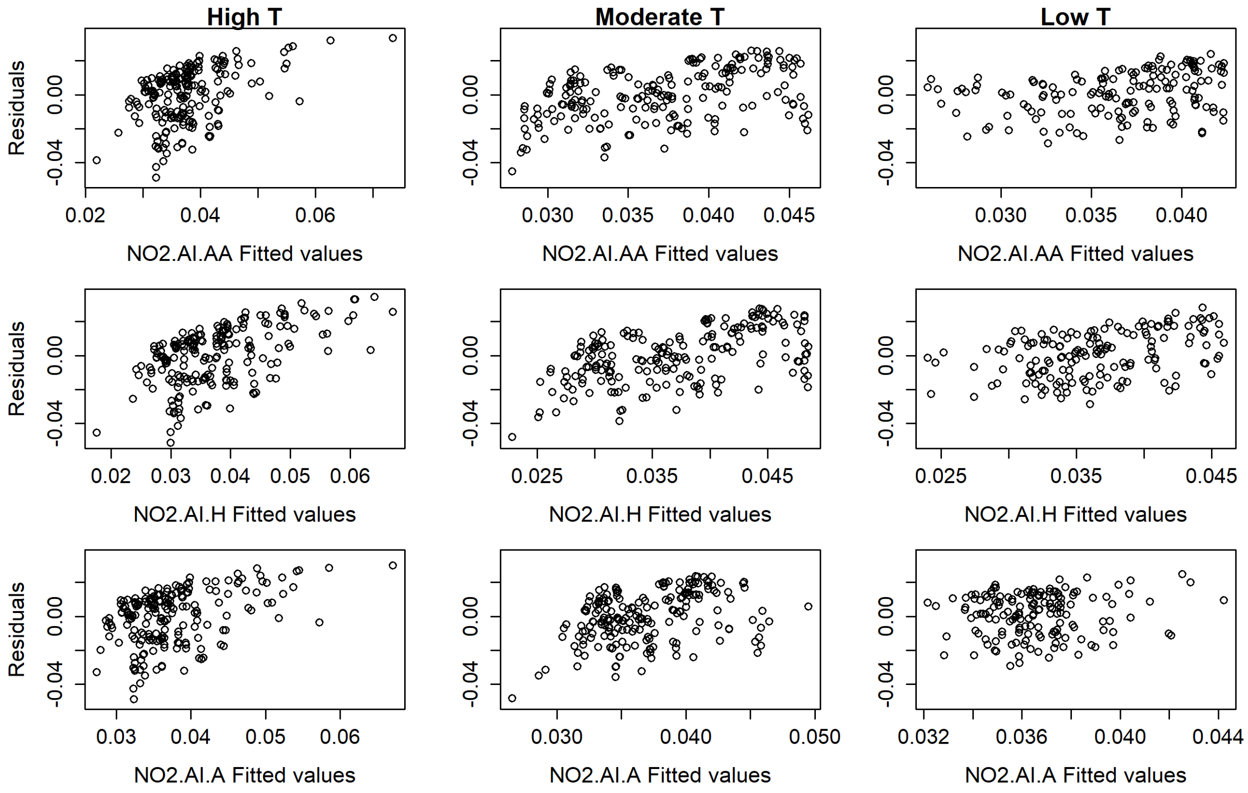

Figure S12. Homogeneity of GAMs for MSA-level mean MDA8 O_3 and NO_2 inequalities for Black and African Americans (top row), Hispanics and Latinos (second row), and Asians (third row) using the spline smoother in the 'gam' package.

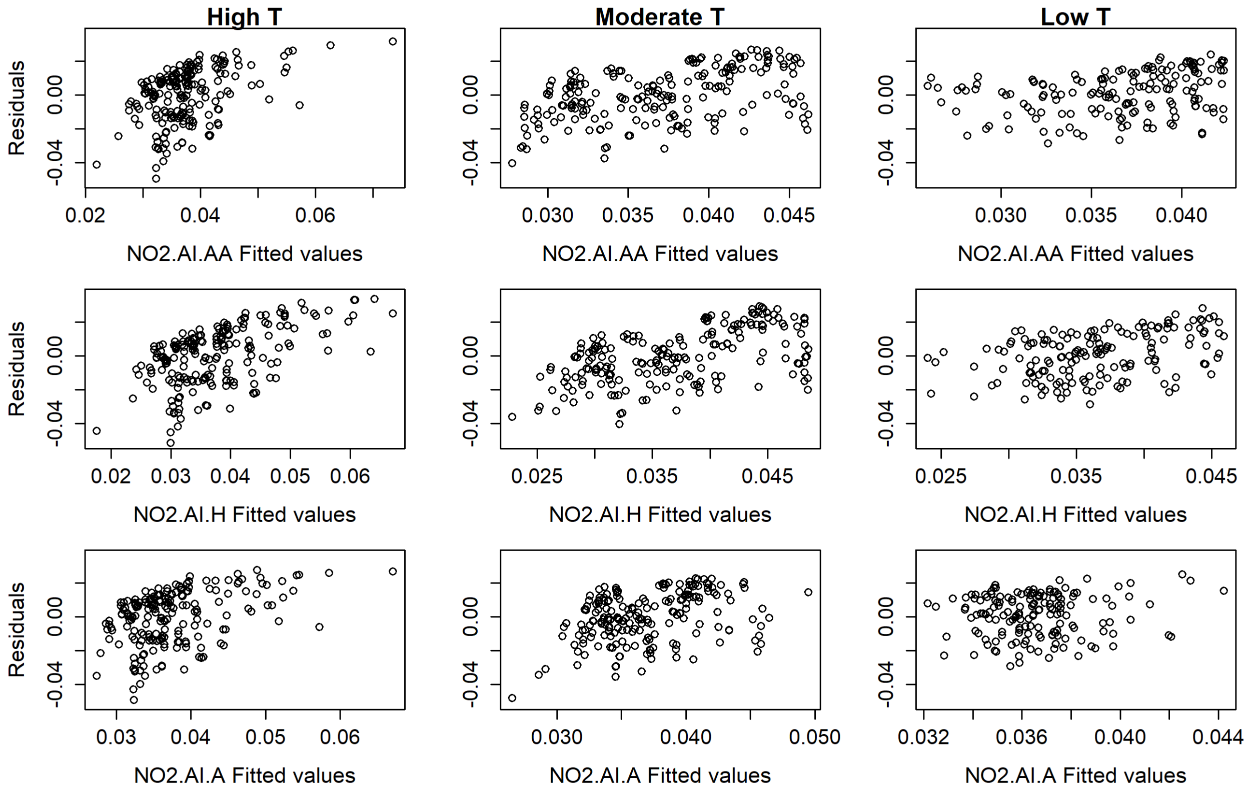

Figure S13. Homogeneity of GAMs for MSA-level mean MDA8 O_3 and NO_2 inequalities for Black and African Americans (top row), Hispanics and Latinos (second row), and Asians (third row) using cubic spline smoother in 'mgcv' package.

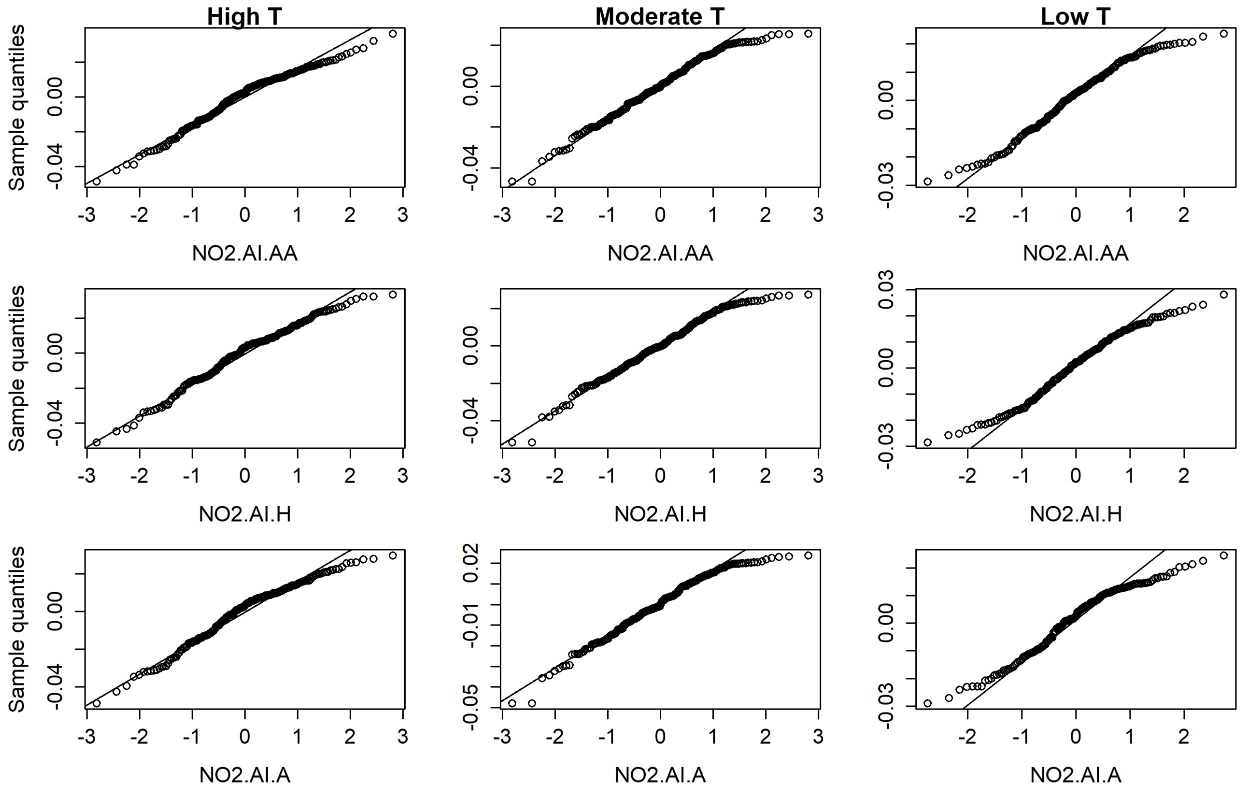

Figure S14. Normality of GAMs for MSA-level mean MDA8 O_3 and NO_2 inequalities for Black and African Americans (top row), Hispanics and Latinos (second row), and Asians (third row) using LOESS in 'gam' package.

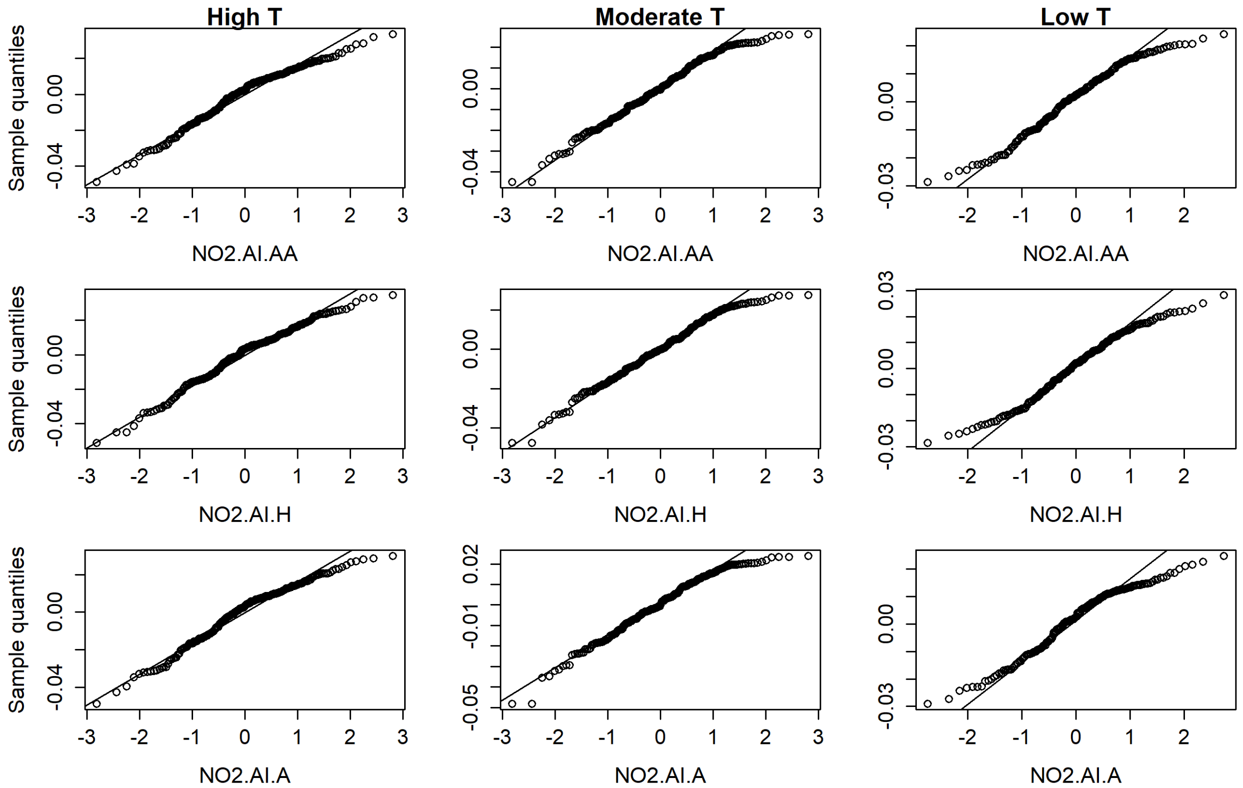

Figure S15. Normality of GAMs for MSA-level mean MDA8 O_3 and NO_2 inequalities for Black and African Americans (top row), Hispanics and Latinos (second row), and Asians (third row) using spline smoother in 'gam' package.

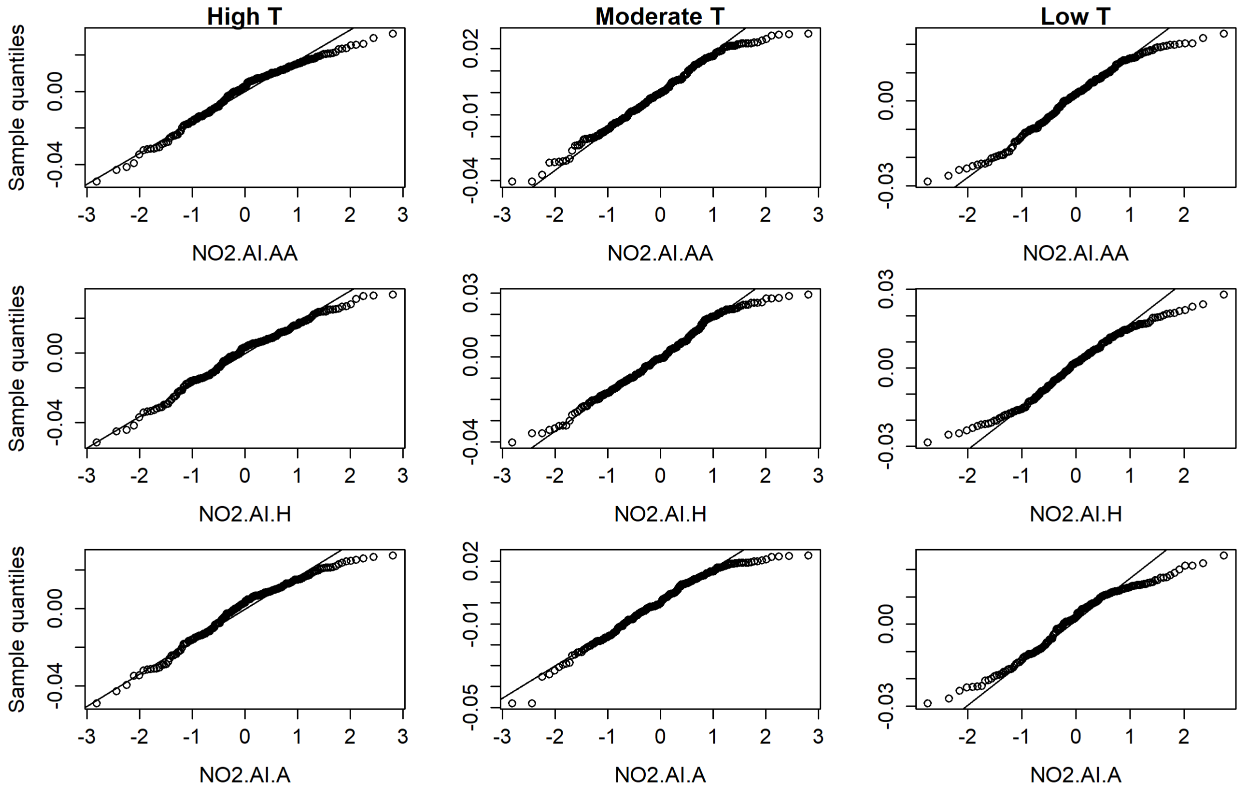

Figure S16. Normality of GAMs for MSA-level mean MDA8 O_3 and NO_2 inequalities for Black and African Americans (top row), Hispanics and Latinos (second row), and Asians (third row) using cubic spline smoother in ‘mgcv’ package.

References

1. Pereira, D.; Wind Rose; The MathWorks, Inc.: *MATLAB Central File Exchange*, 2021. https://www.mathworks.com/matlabcentral/fileexchange/47248-wind-rose (accessed November 2021).
2. Hastie, T.; Tibshirani, R. Generalized Additive Models. In *Wiley StatsRef: Statistics Reference Online*.
3. Zuur, A. F.; Ieno, E. N.; Walker, N.; Saveliev, A. A.; Smith, G. M. *Mixed Effects Models and Extensions in Ecology with R*; Springer, 2009. doi:10.1007/978-0-387-87458-6.
4. Hastie, T.; Tibshirani, R.; Friedman, J. *The Elements of Statistical Learning*; Springer New York Inc., 2001.
5. Hastie, T. Generalized Additive Models: Package 'gam'. 1.22-2 ed.; CRAN, 2023.
